# Aberrant neuronal activity-induced signaling and gene expression in a mouse model of RASopathy

**DOI:** 10.1371/journal.pgen.1006684

**Published:** 2017-03-27

**Authors:** Franziska Altmüller, Santosh Pothula, Anil Annamneedi, Saeideh Nakhaei-Rad, Carolina Montenegro-Venegas, Eneko Pina-Fernández, Claudia Marini, Monica Santos, Denny Schanze, Dirk Montag, Mohammad R. Ahmadian, Oliver Stork, Martin Zenker, Anna Fejtova

**Affiliations:** 1 RG Presynaptic Plasticity, Leibniz Institute for Neurobiology, Magdeburg, Germany; 2 Institute for Human Genetics, Otto von Guericke-University, Magdeburg, Germany; 3 Department of Neurochemistry and Molecular Biology, Leibniz Institute for Neurobiology, Magdeburg, Germany; 4 Institute of Biochemistry and Molecular Biology II, Medical Faculty of the Heinrich Heine University, Düsseldorf, Germany; 5 Department of Genetics & Molecular Neurobiology, Institute of Biology, Otto-von-Guericke-University Magdeburg, Germany; 6 Neurogenetics Special Laboratory, Leibniz Institute for Neurobiology, Magdeburg, Germany; 7 Center for Behavioral Brain Science, Otto von Guericke University, Magdeburg, Germany; 8 Department of Psychiatry and Psychotherapy, University Hospital, Friedrich-Alexander-University Erlangen-Nuremberg, Erlangen, Germany; Florey Institute of Neuroscience and Mental Health, AUSTRALIA

## Abstract

Noonan syndrome (NS) is characterized by reduced growth, craniofacial abnormalities, congenital heart defects, and variable cognitive deficits. NS belongs to the RASopathies, genetic conditions linked to mutations in components and regulators of the Ras signaling pathway. Approximately 50% of NS cases are caused by mutations in *PTPN11*. However, the molecular mechanisms underlying cognitive impairments in NS patients are still poorly understood. Here, we report the generation and characterization of a new conditional mouse strain that expresses the overactive *Ptpn11*^*D61Y*^ allele only in the forebrain. Unlike mice with a global expression of this mutation, this strain is viable and without severe systemic phenotype, but shows lower exploratory activity and reduced memory specificity, which is in line with a causal role of disturbed neuronal Ptpn11 signaling in the development of NS-linked cognitive deficits. To explore the underlying mechanisms we investigated the neuronal activity-regulated Ras signaling in brains and neuronal cultures derived from this model. We observed an altered surface expression and trafficking of synaptic glutamate receptors, which are crucial for hippocampal neuronal plasticity. Furthermore, we show that the neuronal activity-induced ERK signaling, as well as the consecutive regulation of gene expression are strongly perturbed. Microarray-based hippocampal gene expression profiling revealed profound differences in the basal state and upon stimulation of neuronal activity. The neuronal activity-dependent gene regulation was strongly attenuated in Ptpn11^D61Y^ neurons. In silico analysis of functional networks revealed changes in the cellular signaling beyond the dysregulation of Ras/MAPK signaling that is nearly exclusively discussed in the context of NS at present. Importantly, changes in PI3K/AKT/mTOR and JAK/STAT signaling were experimentally confirmed. In summary, this study uncovers aberrant neuronal activity-induced signaling and regulation of gene expression in Ptpn11^D61Y^ mice and suggests that these deficits contribute to the pathophysiology of cognitive impairments in NS.

## Author summary

RASopathies are genetic disorders often accompanied by a developmental delay and intellectual disabilities. However, the molecular mechanisms causing cognitive impairments are yet unclear. RASopathies are linked to mutations in components of the Ras/MAPK pathway. This pathway enables cells to adjust the expression of their genes in response to various environmental stimuli. In the brain, neurodevelopmental signals and neuronal activity are the main modulators of Ras/MAPK signaling, which regulates the expression of neuronal genes. This process is of key importance for normal cognition, learning and memory formation. In this study, we show that neurons expressing a RASopathy-related mutation fail to modulate their gene expression in response to neuronal activity. Paradoxically, despite of an increased basal Ras/MAPK signaling the induction of gene expression by neuronal activity is largely attenuated suggesting the emergence of compensatory mechanisms that dampen the unconstrained signaling. We propose that these mechanisms interfere with a normal induction of Ras/MAPK signaling and subsequent gene expression by neuronal activity. These findings have a therapeutic relevance, as they suggest that possible treatment strategies should focus on the restoration of the physiological dynamics of neuronal Ras/MAPK signaling rather than on a general dampening of this cascade.

## Introduction

Noonan syndrome (NS) is a congenital developmental disorder characterized by reduced growth, craniofacial abnormalities, heart defects and variable cognitive deficits [1]. NS belongs to the RASopathies, a group of genetic diseases linked to mutations in genes coding for components or regulators of the Ras/mitogen-activated protein/extracellular signal-regulated kinase (Ras/MAPK) signaling cascade [2]. The dysregulation of this signaling pathway is believed to cause the pleiotropic phenotype associated with NS. However, the molecular mechanisms underlying the manifestations in particular organ systems are still poorly understood [1]. In more than 50% of cases NS is caused by mutations in *PTPN11*, encoding a widely expressed non-receptor protein tyrosine phosphatase (also known as SHP2) that dephosphorylates a broad range of cellular substrates [3] among them the small GTPase Ras [4]. The most prominent cellular role of the Ptpn11 is the regulation of the pleiotropic Ras/MAPK and phosphoinositide-3 kinase (PI3K) cascades initiated classically upon growth factor or hormone binding to their transmembrane receptor kinases. While Ptpn11 mostly facilitates Ras/MAPK signaling [5,6], both positive and negative regulatory effects have been demonstrated on PI3K signaling [7]. NS-associated mutations in *PTPN11* typically lead to an increased phosphatase activity of the enzyme [8–10], and in line with the positive regulatory role of Ptpn11 in Ras/MAPK signaling, this pathway was shown to be hyperactive in NS [10]. An increased phosphorylation of the extracellular signal-regulated kinases 1 and 2 (ERK1/2) was reported for cell lines, neuronal and non-neuronal tissues overexpressing mutants of Ptpn11 leading to a higher phosphatase activity [10–13]. Increased Ras/MAPK signaling seems to play a major role in the pathogenesis of NS-related defects, since several studies demonstrated that the inhibition of this signaling cascade by pharmacological inhibitors of the mitogen-activated protein kinase kinase (shortly called MEK) was able to fully or partially rescue cardiac [14,15] and craniofacial malformations [16], as well as defects in growth hormone release, which likely contribute to growth retardation seen in patients with NS [13].

Ras/MAPK signaling controls cell proliferation and differentiation in non-neuronal cells. In mature neurons, which are terminally differentiated cells, the function of this pathway shifts towards the mediation of multiple forms of neuronal plasticity, which underlie learning and memory formation in animals and in humans [17]. Recently, disturbed Ras/MAPK signaling was suggested to be the cause of impaired learning and defects in hippocampal synaptic transmission and plasticity in animals carrying the overactive *Ptpn11*^*D61G*^ mutation, which is a known mutation causing NS in humans [11]. The level of ERK phosphorylation was increased in the brains of heterozygous *Ptpn11*^*D61G*^ animals. Moreover, the treatment with Ras or MEK inhibitors was able to dampen the ERK hyperphosphorylation. This treatment also reversed learning deficits and normalized synaptic transmission in *Ptpn11*^*D61G*^ mice [11]. However, the neurobiological mechanistic explanation of these findings is not straightforward. Ras/MAPK signaling is normally activated by neuronal activity and therefore it is not clear, why the increased Ras/MAPK activity in neurons expressing *Ptpn11*^*D61G*^ leads to disturbances rather than enhancement of the respective functional readouts.

The main effector of neuronal activity-induced Ras/MAPK signaling is ERK, which is activated by phosphorylation upon neuronal stimulation [18]. ERK controls the trafficking of postsynaptic glutamate receptors, which plays an important role in the activity-induced potentiation of neurotransmission [19]. Phosphorylated ERK1/2 can also translocate from axons and dendrites to the soma and enter the nucleus to mediate neuronal activity-induced reprogramming of gene expression. The activation and translocation of ERK are of key importance for the persistent usage-dependent neuronal plasticity [18]. It is currently unknown to what extent these molecular functions of Ras/MAPK signaling in neurons are affected by NS-associated mutations in *Ptpn11*.

Here, we report the generation and characterization of a new conditional mouse strain, in which the expression of the NS-linked Ptpn11^D61Y^ 1) occurs from an endogenous locus modeling precisely the situation in patients, and 2) is restricted to the excitatory neurons of the forebrain excluding influence of any systemic phenotype. We used this model to investigate effects of Ptpn11^D61Y^ expression on behavior, neuronal morphogenesis, cellular signaling, and neuronal-activity induced regulation of gene expression.

## Results

### Generation of the mouse strain expressing Ptpn11^D61Y^ in the forebrain

**Fig 1:**
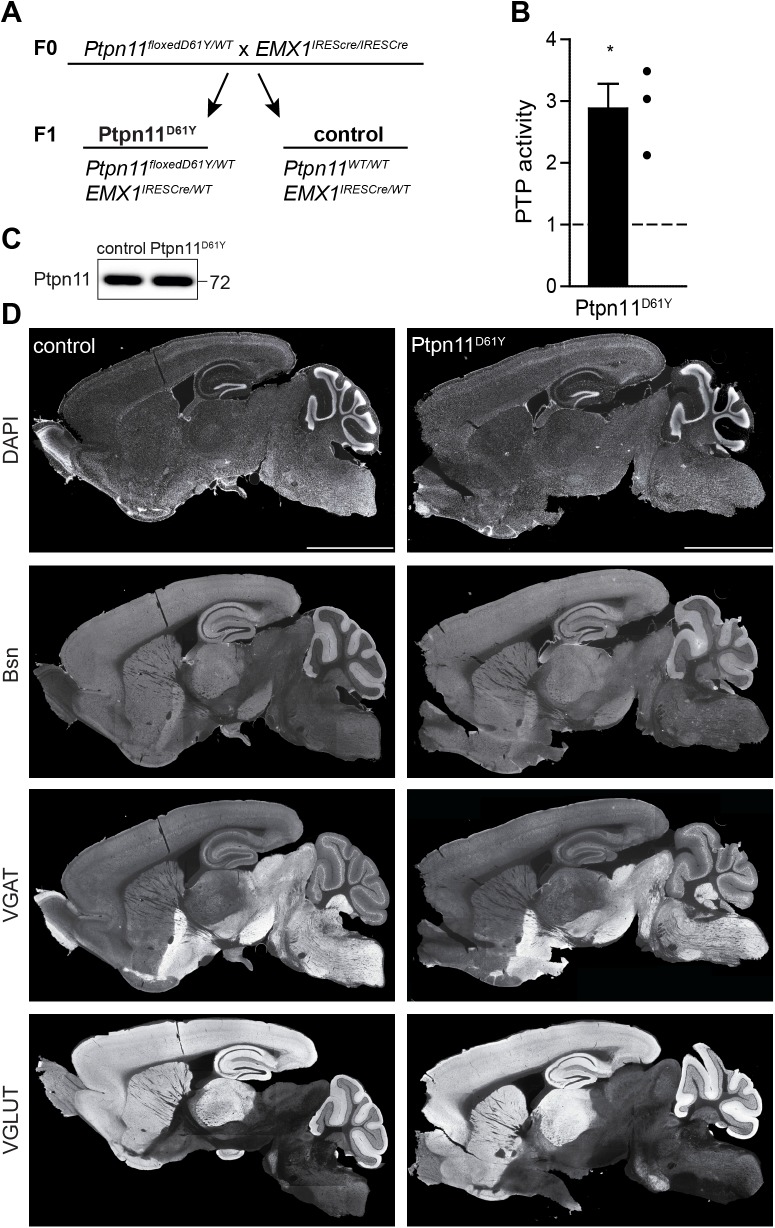
Establishment of *Ptpn11*^*D61Y*^ mouse strain. **(A)** Breeding scheme showing genotypes of parental (F0) and offspring (F1) generation. Progeny segregated in 2 genotypes used as mutants (Ptpn11^D61Y^) and controls (control) in all experiments. **(B)** Ptpn11-specific PTP activity was 3-fold higher in Ptpn11^D61Y^ forebrain homogenate as compared to controls. Data are shown as normalized mean ± SEM and analyzed using one sample t-test, *p<0.05. Dots indicate the values of 3 Ptpn11^D61Y^ animals. **(C)** The expression of Ptpn11 is not altered in the forebrain of Ptpn11^D61Y^ mice. **(D)** The overall brain morphology is not affected in Ptpn11^D61Y^ animals. Sagittal sections of brains from control and Ptpn11^D61Y^ mice were stained with antibodies against Bsn, VGAT, and VGLUT and with DAPI. Scale bar: 3.5 mm.

To obtain viable animals expressing the overactive *Ptpn11*^*D61Y*^ allele from an endogenous locus selectively in the brain, we crossed heterozygous conditional *Ptpn11*^*floxedD61Y/WT*^ animals [20] with animals homozygous for *Emx1*^*IREScre*^ allele (Fig 1A). The endogenous *Emx1* promoter drives the expression of the cre recombinase from embryonic day 10.5 onwards selectively in excitatory neurons and astrocytes of the forebrain and ventral pallium in mice carrying the *Emx1*^*IREScre*^ allele [21]. Our breeding scheme was designed to yield offspring that are all heterozygous for *Emx1*^*IRESCre*^ allele, while 50% are wildtype for the *Ptpn11* locus (Ptpn11^WT/WT^; from here on referred to as control) and 50% are heterozygous for the mutated allele (Ptpn11^floxedD61Y/WT^; referred to as Ptpn11^D61Y^ further on). The analysis of 348 male offspring confirmed an equal ratio of animals surviving by the age of 5 weeks (49% control vs. 51% Ptpn11^D61Y^). Female offspring were not weaned and genotyped but their occurrence at birth did not differ from their male littermates. Thus, the spatially and temporally restricted expression of Ptpn11^D61Y^ obviously circumvented embryonic lethality reported earlier for its constitutive expression [20]. Quantitative immunoblotting revealed no changes in the expression of Ptpn11 in the forebrain of Ptpn11^D61Y^ mice as compared to controls (Fig 1C, n = 3 animals per genotype). To prove the expression of an overactive phosphatase, the protein tyrosine phosphatase (PTP) activity of Ptpn11 immunoprecipitated from forebrain lysates of 8 weeks-old animals was measured. Indeed, the PTP activity was nearly threefold higher in Ptpn11^D61Y^ compared to controls (Fig 1B; 288±4% PTPase activity of control, n = 3 experiments with 1 mouse per genotype; *p≤0.05; one-sample t-test). An examination of sagittal brain slices stained with markers for excitatory and inhibitory synapses did not reveal any gross morphological defects in the brain structure in Ptpn11^D61Y^ animals (Fig 1D).

### Ptpn11^D61Y^ animals show reduced exploratory activity and a deficit in memory specificity

To test the effect of Ptpn11^D61Y^ on forebrain function in vivo, we analyzed the exploratory behavior and memory formation in our mouse model. Intrinsically motivated locomotor activity was tested in the animal’s home cage. The hour-wise comparison confirmed a significant time effect on this activity (12 hours dark vs. 12 hours light; two-way repeated measures ANOVA: F_23,92_ = 25.61, p<0.0001), indicating a normal circadian pattern in Ptpn11^D61Y^ and control animals. There was a slight effect of genotype during the overall time analyzed (two-way repeated measures ANOVA: F_1,4_ = 7.84, p = 0.049). However, Bonferroni post hoc tests revealed no significant differences at any given time point. The mean values of activity during dark or light phase were not changed (12 hours dark: control, 48±4% activity, n = 12; Ptpn11^D61Y^, 46±5% activity, n = 10; during 12 hours light: control: 20±2% activity; Ptpn11^D61Y^: 25±6% activity). There was no significant interaction of time and genotype (two-way repeated measures ANOVA: F_23,92_ = 1.46, p>0.05). Of note, no differences in the intrinsically motivated activity were observed between 9:00 a.m. and 5:00 p.m. when further behavioral tests were conducted.

**Fig 2:**
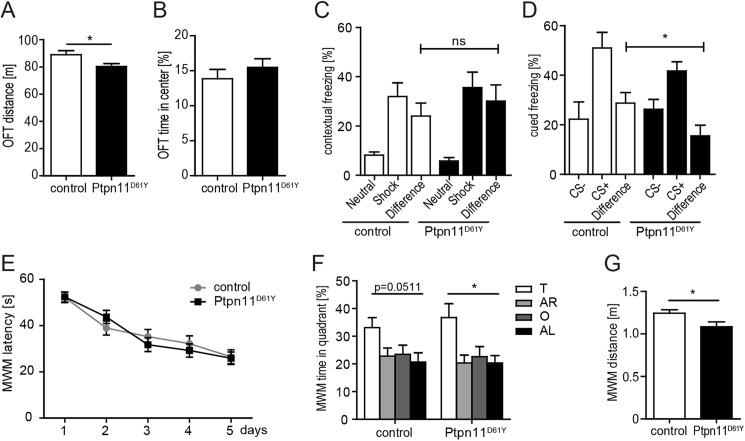
Ptpn11^D61Y^ animals show reduced exploratory activity and mild impairment of memory specificity. **(A)** Ptpn11^D61Y^ animals show a reduced run distance in the OFT. **(B)** The time spent in the center of field did not differ in the OFT. **(C)** Ptpn11^D61Y^ animals display normal levels of contextual fear memory. **(D)** In cued fear conditioning the differentiation of CS+ and CS- was reduced in the mutants compared to controls. **(E, F)** In the MWM, the escape latency during training **(E)** as well as the time spent in the target quadrant (T) in the first probe trial (F) are comparable between the genotypes indicating normal spatial memory in Ptpn11^D61Y^ (AR, adjacent right; O, opposite; AL adjacent left quadrant). **(G)** Ptpn11^D61Y^ animals swam shorter distances in the probe trial. Data are shown as mean ± SEM analyzed using unpaired t-test, one-way ANOVA or two-way repeated measures ANOVA (n = 10–12; *p<0.05).

Next we measured the exploratory behavior in the open field task (OFT) providing a novel environment. Ptpn11^D61Y^ mice showed a reduced exploratory activity in OFT, indicated by a shorter distance run compared to littermate control mice (Fig 2A; control: 89±3 m, n = 12; Ptpn11^D61Y^: 80±2 m, n = 12; unpaired t-test, p<0.05). However, the time spent in the center did not differ between the genotypes (Fig 2B; control, 14±1% of center time, n = 12; Ptpn11^D61Y^, 15±1% of center time, n = 12; unpaired t-test, p>0.05).

Furthermore, we investigated the behavior in a combined cued/contextual fear conditioning task. Both genotypes (n = 10 Ptpn11^D61Y^, n = 10 control) showed comparable freezing levels in the shock context (initial 2 min of the test session: control, 32±6% freezing; Ptpn11^D61Y^, 35±6% freezing; total 8 min of the test session: control, 29±5% freezing; Ptpn11^D61Y^, 30±4% freezing) and a clear discrimination from the unconditioned control context (Fig 2C; differences of shock context and neutral context: control, 24±5% freezing; Ptpn11^D61Y^, 30±7% freezing). By contrast, during auditory cued retrieval the mutant mice showed a reduced ability to discriminate CS+ and CS- (Fig 2D; differences: control, 29±4% freezing, n = 10; Ptpn11^D61Y^, 16±4% freezing, n = 10; unpaired t-test, p<0.05). The response to the CS+ was slightly reduced (control, 51±6% freezing; Ptpn11^D61Y^, 41±4% freezing), while that to the CS- was somewhat increased (control, 22±7% freezing; Ptpn11^D61Y^, 26±4% freezing).

Finally we assessed spatial learning and memory in the Morris water maze (MWM) task. Both the Ptpn11^D61Y^ (n = 12) and control (n = 12) mice performed in a comparable manner and successfully learned the location of the submerged platform. During training, escape latency did not differ between the genotypes (Fig 2E; two-way repeated measures ANOVA: F_1,94_ = 0.05, p>0.05), however, Ptpn11^D61Y^ mice had a reduced path length (two-way repeated measures ANOVA: F_1,94_ = 3.96, p<0.05;). Lower average speed of Ptpn11^D61Y^ mice (repeated measures ANOVA F_(1,94)_ = 26.773, p<0.0001) explains escape latencies similar to controls despite slightly reduced path length. During probe trials, mice of both genotypes spent more time in the target quadrant (Fig 2F; control: 33±4%; Ptpn11^D61Y^: 37±5%) when compared to non-target quadrants indicating spatial memory (control, one-way ANOVA, probe trial 1: F_3,44_ = 2.798, p>0.05, probe trial 2: F_3,44_ = 4.302, p<0.01; Ptpn11^D61Y^, one-way ANOVA, probe trial 1: F_3,44_ = 4.683, p<0.01, probe trial 2: F_3,44_ = 5.606, p<0.01). Interestingly, the total distance travelled (Fig 2G; control, 1.243±0.41 m; Ptpn11^D61Y^, 1.084±0.59 m, n = 12; one-way ANOVA, F_(,22)_ = 4.969, unpaired t-test, p<0.05;) during probe trial 1 in the MWM was also reduced in Ptpn11^D61Y^ mice. In summary Ptpn11^D61Y^ mice displayed lower exploratory activity and reduced memory specificity, which is in line with a causal role of disturbed neuronal Ptpn11 signaling in the development of NS-linked cognitive deficits.

### Morphometric analyses of Ptpn11^D61Y^ neurons

**Fig 3:**
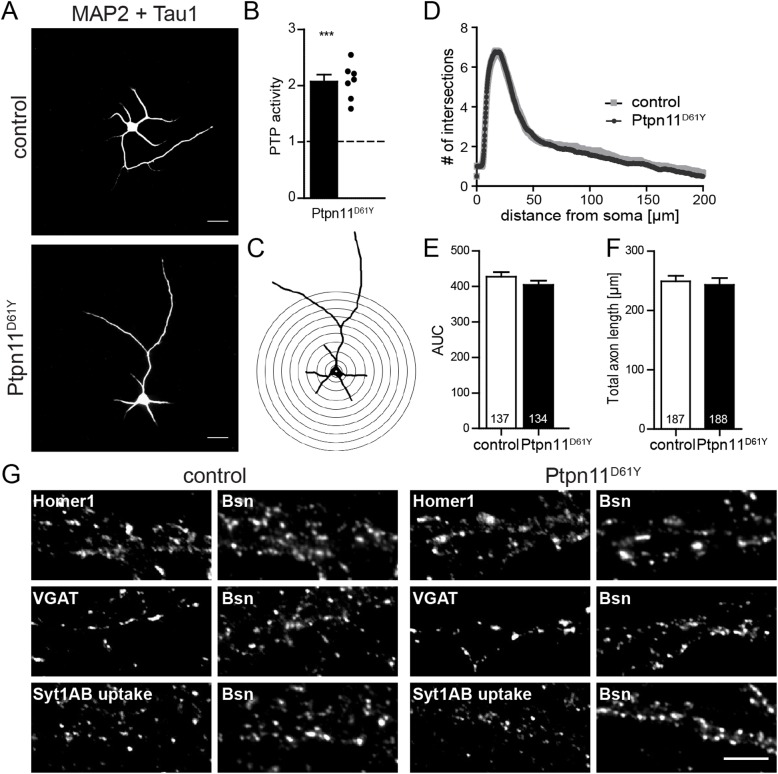
Morphometric analysis of Ptpn11^D61Y^ neurons. **(A)** DIV5 neurons were stained with antibodies against the dendritic marker MAP2 and the axonal marker Tau1 for the assessment of dendritic arborization and axonal outgrowth. Scale bar: 50 μm. **(B)** The Ptpn11-specific PTP activity showed a 2-fold increase in cultured cortical Ptpn11^D61Y^ neurons compared to controls. The points indicate the values from cultures derived from individual animals. **(C)** Example image of a neuron from (A) after binarization and placement of the array of concentric circles used for Sholl analysis. **(D)** Sholl analysis of DIV5 neurons is shown, where the number of ramifications is plotted against the distance from the soma. **(E, F)** The area under the curve (AUC) in Sholl analysis **(E)** and the axonal length **(F)** do not differ between control and Ptpn11^D61Y^ neurons. **(G)** Immunostaining of excitatory (positive for Homer1), inhibitory (positive for VGAT) and active (showing the syt1AB uptake) synapses in DIV14 neurons from control and Ptpn11^D61Y^. Scale bar: 5 μm. Data are presented as mean ± SEM and analyzed using unpaired t-test. The numbers in columns indicate the number of cells analyzed.

Since RASopathies are neurodevelopmental disorders [22] and since there is evidence that Ptpn11 is required for neuronal outgrowth [23], we tested whether the expression of Ptpn11^D61Y^ affects neuronal morphology and synaptogenesis. To this end, we analyzed axonal outgrowth and dendritic arborization in cultured hippocampal neurons derived from Ptpn11^D61Y^ newborns and their control littermates. Cultured cortical neurons from mutants showed a 2-fold increase in the Ptpn11-specific PTP activity, which confirms the transgene activation also in neurons grown in vitro (Fig 3B; control vs. Ptpn11^D61Y^ 208±12% of control PTPase activity, n = 5 vs. 7, unpaired t-test, p≤0.0001). Dendrites and axons of neurons cultured for 5 days in vitro (DIV) were visualized by immunostaining with antibodies against MAP2 and Tau1, respectively (Fig 3A). The dendritic arborization was assessed by counting the number of intersections on the array of concentric circles centered over the cell body (Fig 3C) and plotting the number of intersection as a function of distance from the soma (Sholl analysis; Fig 3C and 3D). No significant differences were found in this analysis (Fig 3D and 3E; control vs. Ptpn11^D61Y^, 427.5±13 vs. 404.5±13, n = 137 vs. 134 cells from 3 experiments, p≥0.05; unpaired t-test). The length of the longest outgrowing neurite did also not differ between the genotypes (Fig 3F; control vs. Ptpn11^D61Y^, 249±9 vs. 243±11 μm, n = 187 vs. 188 cells from 3 experiments, p≥0.05, unpaired t-test). Thus, the Ptpn11^D61Y^ mutation does not lead to profound changes in the neuronal morphology *in vitro*, which is consistent with the unchanged gross brain morphology in these mice (Fig 1G).

Next we tested whether the neuronal expression of Ptpn11^D61Y^ affects synaptogenesis. Synapses were quantified within 20 μm long segments of proximal dendrites. Excitatory synapses were identified as puncta co-labeled with the presynaptic marker Bassoon (Bsn) and the marker of excitatory postsynapses Homer1. Inhibitory synapses were labeled for the markers vesicular GABA transporter (VGAT) and Bsn. The number of excitatory and inhibitory synapses was not altered, comparing control and Ptpn11^D61Y^ neurons (Fig 3G; control vs. Ptpn11^D61Y^; excitatory: 46±2 vs. 48±2 puncta, n = 183 vs. 185 cells from 11 experiments; inhibitory: 17±2 vs. 16±1 puncta, n = 49 vs. 47 cells from 3 experiments). Usually, a significant fraction of synapses are functionally silent despite their normal morphological appearance [24]. To visualize active presynapses undergoing evoked neurotransmitter release, we depolarized living neurons by a brief application of 50 mM KCl in the presence of an antibody against the luminal epitope of synaptotagmin1 (Syt1 AB). This antibody binds to its epitope when exposed to the synaptic cleft during synaptic vesicle fusion and is taken up during compensatory endocytosis [25]. We did not observe significant differences in the density of active presynaptic boutons between the genotypes (Fig 3G; 13±1 vs. 12±1 puncta per 20 μm dendrite, n = 82 vs. 61 cells from 5 experiments). Altogether these experiments suggest a normal synaptogenesis in Ptpn11^D61Y^ neurons.

### Surface expression of synaptic glutamate receptors is altered in Ptpn11^D61Y^ neurons

**Fig 4:**
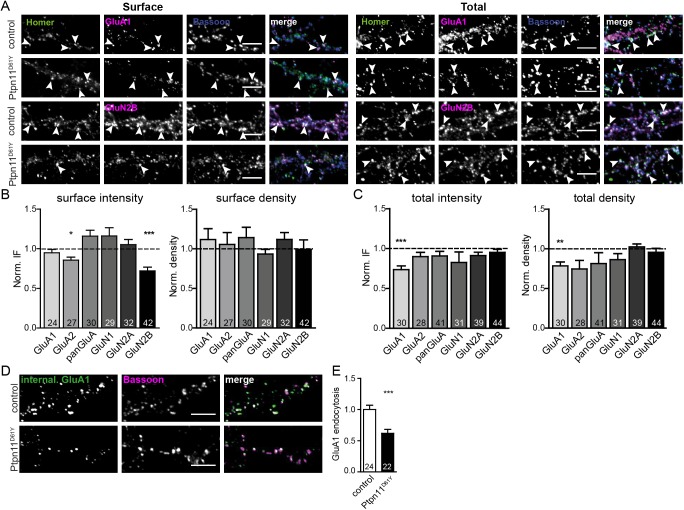
Synaptic expression and surface trafficking of glutamate receptors is affected in Ptpn11^D61Y^. **(A)** Representative examples of the staining of synaptic surface (left) and total (right) glutamate receptors containing GluA1 or GluN2B subunit in neurons from control and from Ptpn11^D61Y^. Excitatory synapses were stained for Homer1 and Bassoon. **(B, C)** Synaptic IF intensity and density (puncta per 20 μm of proximal dendrite) of surface **(B)** and total **(C)** staining for all tested receptor subunits are shown. Values are normalized to the respective value in control, represented by the dashed line in the graphs. Note the reduced surface expression of GluA2 and GluN2B and the decrease of the overall expression of GluA1 in Ptpn11^D61Y^ neurons as compared to controls. All numerical values and statistics are listed in S1 Table. **(D, E)** The rate of internalization of the GluA1-containing AMPARs is reduced in Ptpn11^D61Y^ cells. Scale bars: 5 μm. Data are presented as mean ± SEM and analyzed using unpaired t-test (*p≤0.05, **p≤0.01, ***p≤0.001). Numbers in columns indicate the number of cells analyzed.

Ras signaling has been implicated in the regulation of the delivery of AMPARs to the postsynaptic plasma membrane [26], which is a major determinant of the sensitivity of postsynapses to the neurotransmitter glutamate. To test a possible dysregulation of AMPAR trafficking in Ptpn11^D61Y^ neurons, we quantified the overall synaptic expression of AMPARs (total) and the fraction of AMPARs residing on the surface of spines (surface). In fact only surface receptors can be activated by neurotransmitter and thus represent the fraction contributing to synaptic transmission. To this end, we stained hippocampal neurons prepared from control and Ptpn11^D61Y^ animals with an antibody binding to the extracellular epitopes of all AMPAR isoforms 1–4 (panGluA) and antibodies specific for the subunits GluA1 and GluA2 (Fig 4A and 4B, and S1 Fig; see S1.1 and S1.2 Tables for all values). Receptors on the surface were visualized by the incubation of living cells with the respective antibody followed by fixation and imaging. To quantify the total synaptic expression of AMPARs immunostaining was done on fixed cells. There were no changes in the number of synapses showing surface immunoreactivity for any tested antibody comparing Ptpn11^D61Y^ to control neurons (Fig 4C and S1 Fig). Nevertheless, our quantification revealed a slight decrease in the intensity of the GluA2 surface staining in Ptpn11^D61Y^ neurons (Fig 4B and S1 Fig; 86±4% of controls, p<0.05, unpaired t-test). Regarding the total synaptic expression of AMPARs we observed no significant differences using panGluA or GluA2 antibodies. However, the number of stained synapses and their staining intensity for GluA1 were decreased by about 25% in Ptpn11^D61Y^ neurons (Fig 4A and 4C and S1 Fig). The unchanged surface expression of GluA1 in spines showing lower total expression argues for less efficient endocytosis of this subunit, which was tested in the following experiment. The surface fraction of GluA1 receptors was labeled using a specific antibody in living cells incubated at 4°C to transiently block membrane trafficking. Then, the endocytosis was monitored upon the release of temperature block by immunostaining as described previously [27]. In this assay, we observed less internalized GluA1 in Ptpn11^D61Y^ neurons (Fig 4D; 62%±6 of control, p<0.01, unpaired t-test) supporting an aberrant trafficking of GluA1-containing AMPARs in spines of neurons expressing the *Ptpn11*^*D61Y*^ allele.

Next we tested potential changes in the expression of N-methyl-D-aspartate receptors (NMDARs) in Ptpn11^D61Y^ neurons. The total expression levels and the number of stained synapses were unchanged for GluN1, GluN2A and GluN2B subunits of NMDARs (for all numerical values see S1.1 and S1.2 Tables). However, we observed a significant decrease of the GluN2B-subunit containing receptors on the surface of Ptpn11^D61Y^ neurons (Fig 4A–4C and S1 Fig; 72±5% of control, p<0.001, unpaired t-test). In summary, these data reveal notable changes in the regulation of the surface expression of synaptic AMPARs and NMDARs in Ptpn11^D61Y^ neurons and suggest their aberrant trafficking as a potential underlying mechanism.

### Activity-induced phosphorylation of ERK

**Fig 5:**
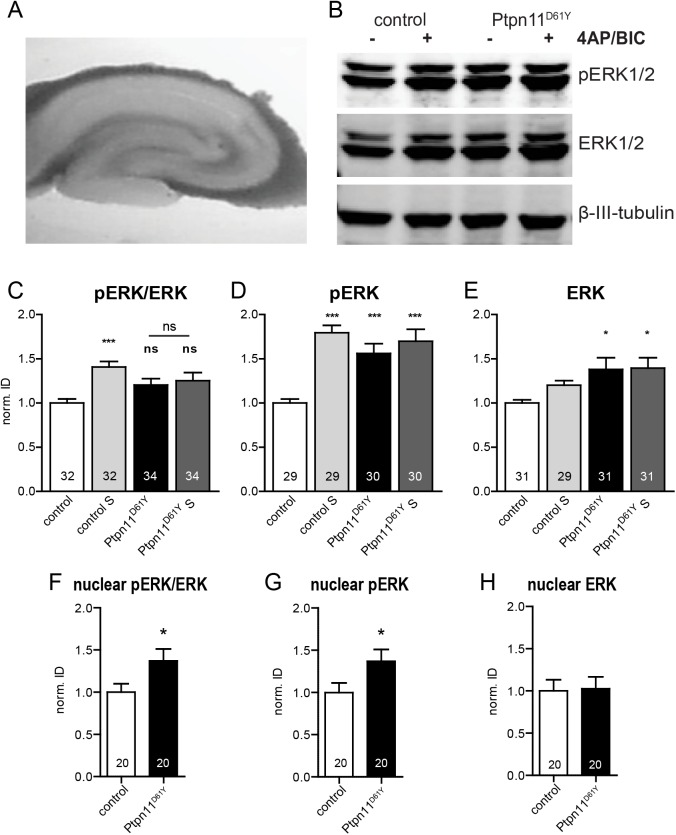
Neuronal activity-induced phosphorylation of ERK is disturbed in Ptpn11^D61Y^. **(A)** Exemplary photograph of an acute slice used for experiments. **(B)** Western blot of lysates from control and Ptpn11^D61Y^ acute slices treated with 4AP/Bic (**+**) or vehicle (**-**) for 10 minutes and probed with antibodies against pERK, ERK and β-III-tubulin. The latter was used as a loading control. **(C-E)** Quantification of the Western blot experiment as exemplified in B is shown. The stimulation of control slices led to a significant increase of the pERK level **(C, D)**. The basal pERK level was increased in Ptpn11^D61Y^ slices compared to controls. No further increase of pERK immunoreactivity could be detected upon stimulation of neuronal activity **(C, D)**. Note the increased total expression of ERK in the slices from Ptpn11^D61Y^ in the basal state and upon stimulation **(E)**. **(F-H)** The quantification of ERK phosphorylation in the nuclear fraction prepared from forebrains indicates an increase in the pERK level in the samples from Ptpn11^D61Y^ animals as compared to controls. Data are shown as mean ± SEM and analyzed using either one-way ANOVA followed by Bonferroni´s multiple comparison test or unpaired t-test (*p≤0.05, ***p≤0.001). The number of replicates from a total of three independent experiments is indicated in the columns of the graph.

To investigate, how the expression of the overactive *Ptpn11*^*D61Y*^ allele affects the basal and neuronal activity-induced Ras/MAPK signaling, we assessed the phosphorylation of the main downstream target of this pathway, ERK, in acute hippocampal slices from 8 to 11 weeks old Ptpn11^D61Y^ animals and their control siblings. Slices were treated with vehicle or 2.5 mM 4-aminopyridine (4AP) and 50 μM Bicuculline (Bic) for 10 min. This treatment is well established to rapidly enhance neuronal activity and leads to ERK phosphorylation [28]. Immediately after the treatment slices were collected, tissue lysates were prepared and processed for analyses by quantitative immunoblots (Fig 5A and 5B). The expression of total ERK and its phosphorylated active form (pERK) was measured and the ratio of pERK/ERK was calculated. The basal pERK level was significantly increased in Ptpn11^D61Y^ samples as compared to controls (Fig 5D; 156±11% of control). The total ERK expression in Ptpn11^D61Y^ samples was also increased but to a lesser extent (Fig 5E; 138±13% of control). As a consequence, the average ratio of the basal pERK/ERK was slightly increased in mutant slices (Fig 5C; 120±7% of control), but this difference did not reach statistical significance. The stimulation of synaptic activity induced a significant rise of pERK levels in controls (Fig 5D; 179±8% of control basal levels), but completely failed to do so in Ptpn11^D61Y^ slices. Here, the pERK levels rose only to 109±8% compared to the basal activity level in the mutant and correspond to 170±14% of the basal activity level in control slices (Fig 5D). The total ERK level was not significantly changed after the induction of activity compared to the basal levels in any of both genotypes (Fig 5E, control vs. Ptpn11^D61Y^: 120±5% vs. 101±9% of basal levels in respective genotype). Correspondingly, the induction of neuronal activity increased the pERK/ERK ratio significantly only in controls but not in Ptpn11^D61Y^ slices (Fig 5C, 141±6% vs. 104±8% of basal activity in the respective genotype). This analysis indicates an aberrant neuronal activity-induced ERK signaling in Ptpn11^D61Y^ mice.

Increased neuronal activity induces ERK phosphorylation and its subsequent translocation to the soma, where it is transported into the nucleus. This mechanism is of key importance for ERK-mediated control of neuronal activity-regulated gene expression [18,29]. To investigate the nuclear pERK levels we isolated nuclear fractions from forebrains of Ptpn11^D61Y^ and control animals and analyzed them in quantitative immunoblots. The nuclear pERK level was significantly increased in Ptpn11^D61Y^ compared to controls (Fig 5G; 137±14% of control), while the total nuclear ERK level was unchanged (Fig 5H; 103±14% of control). Consequently the ratio of nuclear pERK/ERK was higher in Ptpn11^D61Y^ samples (Fig 5F; 137±14% of control), indicating changes in nuclear pERK upon expression of overactive Ptpn11^D61Y^.

**Fig 6:**
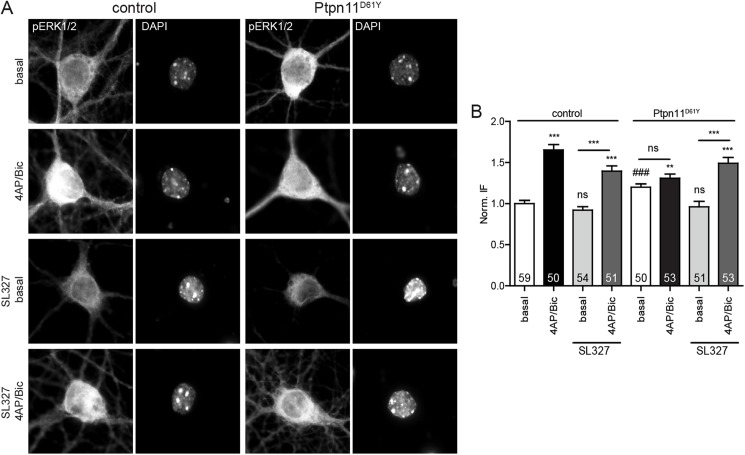
Activity-induced increase of pERK is abolished in Ptpn11^D61Y^ neurons and can be restored upon MEK1 inhibition. Staining **(A)** and quantification **(B)** of pERK in nuclei (marked by DAPI) of excitatory neurons revealed elevated basal nuclear pERK levels in Ptpn11^D61Y^ neurons. The basal levels of nuclear pERK significantly differ between the genotypes (unpaired t-test, ###p≤0.001). The stimulation of neuronal activity induces a rapid increase in the nuclear pERK level (in relation to the basal levels) in control neurons but not in those from Ptpn11^D61Y^ mice. The inhibition of MEK1 using SL327 for 24 h prior to the stimulation normalized the elevated basal pERK in nuclei of Ptpn11^D61Y^ neurons and fully restored the activity-induced increase of nuclear pERK. The identical treatment affected neither the basal nuclear pERK levels nor their stimulation-induced increase in control neurons. Data are presented as mean ± SEM; numbers in columns indicate the numbers of analyzed cells. Significance is assessed using unpaired t-test and one-way ANOVA followed by Bonferroni´s multiple comparison test (**p≤0.01, ***p≤0.001).

To further dissect the effect of the Ptpn11^D61Y^ expression on the activity-induced nuclear translocation of pERK, we moved on to neuronal cultures that allow an easy pharmacological modulation of neuronal activity and an assessment of the subcellular distribution of pERK by immunostaining. The nuclear abundance of pERK in the basal state was increased in 14 DIV old Ptpn11^D61Y^ neurons similarly as it was observed in the nuclear fractions of the forebrain extracts (Figs 5 and 6). Here, the basal nuclear pERK level in Ptpn11^D61Y^ was also increased compared to control (Fig 6; 120±4% of basal control level; p<0.001, unpaired t-test). The treatment with 4AP and Bic for 30 min induced a significant rise in the nuclear pERK level in controls, while there was no further increase in the nuclear pERK in Ptpn11^D61Y^ neurons (Fig 6; control 4AP/Bic: 165±6%, Ptpn11^D61Y^ basal: 120±4%; Ptpn11^D61Y^ 4AP/Bic: 131±5% of basal control level). To test whether this difference results from an increased signaling downstream of Ras, we applied SL327 (1 μM), a potent inhibitor of MEK1, to neurons of both genotypes 24 hours prior to activity induction. The treatment with SL327 neither affected the basal pERK level nor the activity-induced increase in nuclear pERK level in controls (Fig 6; control basal + SL327: 92±4%, control 4AP/Bic + SL327: 140±6%). Importantly, the SL327 treatment fully normalized the nuclear pERK level in Ptpn11^D61Y^ neurons (Ptpn11^D61Y^ basal + SL327: 96±7% of basal control) and restored the activity-induced phosphorylation and nuclear import of ERK in Ptpn11^D61Y^ neurons (Fig 6; Ptpn11^D61Y^ 4AP/Bic + SL327: 149±7% of basal control). These data support the notion that the impaired activity-induced ERK activation and nuclear translocation in Ptpn11^D61Y^ neurons result from an increased activation of Ras/MAPK signaling.

**Fig 7:**
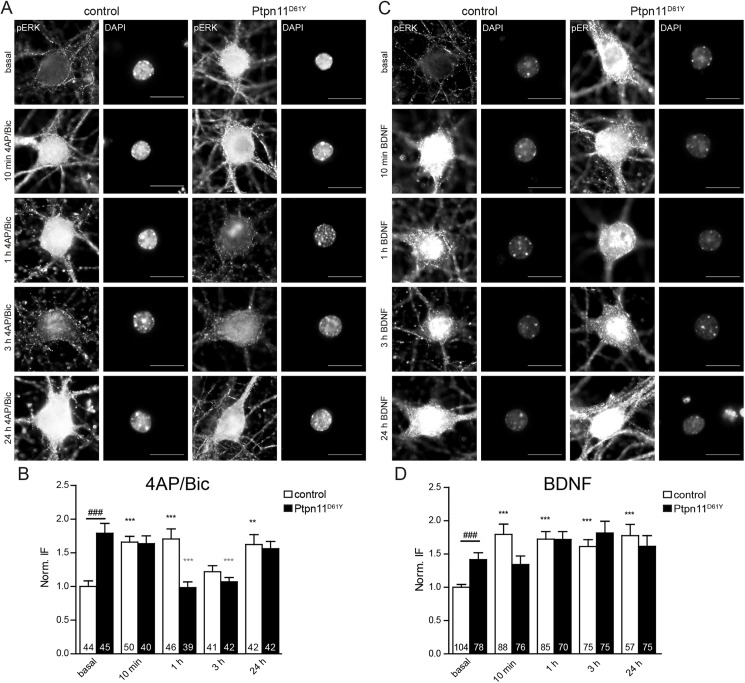
Time course of nuclear pERK accumulation induced by neuronal activity or BDNF is affected in Ptpn11^D61Y^ neurons. **(A, C)** Neurons of both genotypes are stained for pERK at different time points upon 4AP/Bic **(A)** or BDNF **(C)** application. DAPI staining was used as nuclear mask. Scale bar: 10 μm. **(B, D)** Quantification of the nuclear pERK level as exemplified in the staining in **A** and **C**. The basal levels of nuclear pERK significantly differ between the genotypes (unpaired t-test, ### p≤0.001). The time course upon stimulation by both stimuli differed between Ptpn11^D61Y^ and control neurons. Data are shown as mean ± SEM and numbers in columns of graphs indicate number of analyzed cells. The stimulation-induced changes were compared to basal pERK levels in each genotype and significance was assessed using one-way ANOVA and Dunnett´s multiple comparison test (**p≤0.01 and ***p≤0.001) and shown in black for controls and in gray for Ptpn11^D61Y^ neurons.

To further investigate the nuclear translocation of pERK in presence of Ptpn11^D61Y^ we performed time course experiments in cultured hippocampal neurons. We induced the nuclear translocation of pERK either by pharmacological stimulation of neuronal activity using 2.5 mM 4-AP and 50 μM Bic or by application of brain-derived neurotrophic factor (BDNF, 100 ng/μl). BDNF is a natural binding partner of the tropomyosin receptor kinase B (TrkB) that is known to signal via the Ras/MAPK pathway [30]. We quantified the nuclear pERK level 10 min, 1, 3 and 24 h after the onset of the respective treatment (Fig 7). Only excitatory neurons that express the overactive *Ptpn11*^*D61Y*^ allele in our animal model and that were negative for staining against GAD65, a broad marker of inhibitory cells, were considered for this analysis. Both treatments resulted in a rapid increase in nuclear pERK in control, even though the temporal profile of activation differed between the two treatments. Increased neuronal activity induced a biphasic activation profile with an early peak between 10 min and 1 h, followed by a decline of the nuclear pERK level 3 h after the onset of the treatment and a second peak in the nuclear pERK level after 24 h (Fig 7A and 7B; 10 min: 166±8%; 1 h: 171±15%; 3 h: 122±9%; 24 h: 162±15% of basal control, one-way ANOVA). The BDNF treatment showed a fast and persistent increase of the nuclear pERK level at all time points measured (Fig 7C and 7D; 10 min: 180±15%; 1 h: 172±12%; 3 h: 161±10%; 24 h: 177±17% of unstimulated control, one-way ANOVA). In contrast, in Ptpn11^D61Y^ neurons that have an increased nuclear pERK level already in the basal state, both treatments failed to induce a further increase in the nuclear pERK level (Fig 7A and 7B; 4AP/Bic: basal: 179±14%; 10 min: 164±12%; 1 h: 98±9%; 3 h: 107±7%; 24 h: 156±11% of basal control; Fig 7C and 7D; BDNF: basal: 142±10%; 10 min: 134±13%; 1 h: 171±12%; 3 h: 181±18%; 24 h: 161±16% of basal control, one-way ANOVA). Interestingly, 1 h after the induction of neuronal activity a notable drop in the nuclear pERK level was evident in the mutant, while control neurons still showed peak levels of pERK. This suggests that not only the magnitude of ERK phosphorylation and nuclear translocation, but also the temporal dynamics of neuronal Ras/MAPK signaling are disturbed in Ptpn11^D61Y^.

**Fig 8:**
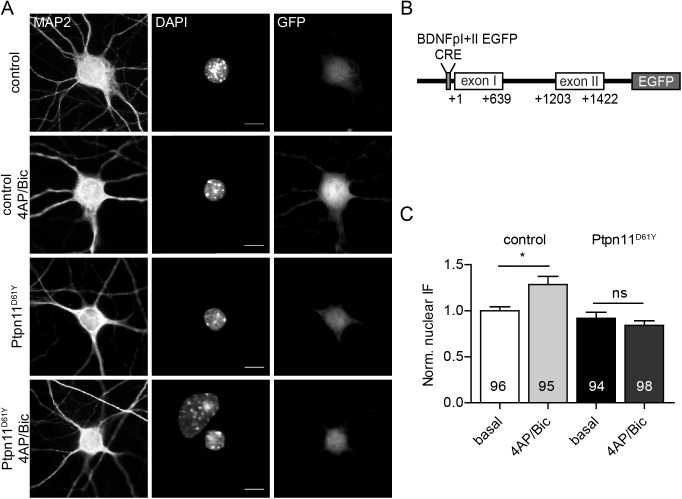
Activity-induced BDNF expression is affected in Ptpn11^D61Y^ neurons. **(A)** Representative images of neurons in low-density cultures infected with the lentivirus containing the BDNFpI+II EGFP reporter in basal conditions and upon treatment with 4AP/Bic for 30 min. Neurons are stained with antibodies against GFP to enhance the intrinsic EGFP signal, MAP2 as a neuronal marker, and DAPI. **(B)** Schema of the activity reporter, in which the BDNF promoters I and II drive the expression of EGFP. The cAMP response element (CRE) is depicted. **(C)** Quantification of the GFP IF that was measured in the nuclei of control and Ptpn11^D61Y^ neurons. The reporter signal increased significantly upon stimulation in control but not in Ptpn11^D61Y^ neurons. Data are shown as mean ± SEM and analyzed using one-way ANOVA followed by Bonferroni´s multiple comparison test (*p≤0.05) Scale bar: 10 μm.

To monitor the pERK-induced gene expression, we used a BDNF promoter activity reporter, in which the BDNF promoters I and II drive the transcription of the EGFP coding sequence (Fig 8A). This promoter region contains a cAMP-response element (CRE), the binding site of the CRE-binding protein (CREB), which is an important downstream nuclear target of nuclear pERK (Fig 8B). We delivered this reporter to neurons from control and Ptpn11^D61Y^ animals grown for 14 DIV in low-density cultures using a viral vector. In these cultures, the basal nuclear pERK levels were comparable in Ptpn11^D61Y^ and control excitatory neurons (S2 Fig, 97±5% of control), which is likely due to very low endogenous network activity in these cultures. Consistently with the results from high-density cultures, the induction of activity using 4AP/Bic increased the nuclear pERK level in control neurons but failed to do so in the neurons from Ptpn11^D61Y^ (S2 Fig; control vs. Ptpn11^D61Y^: 136±8% vs. 99±4% of basal levels in control). We measured the nuclear EGFP fluorescence of the BDNF promoter activity reporter 30 min after the treatment with vehicle (basal conditions) or with 4AP/Bic. The increase in activity led to a notable rise in the expression of the reporter in control cells (Fig 8A and 8C; 129±9% of basal control level), but completely failed to do so in Ptpn11^D61Y^ neurons (84±5% of basal control level), which is in line with the aberrant activity-induced phosphorylation and nuclear translocation of ERK seen in these cells.

### Neuronal activity-controlled gene expression in Ptpn11^D61Y^

**Fig 9:**
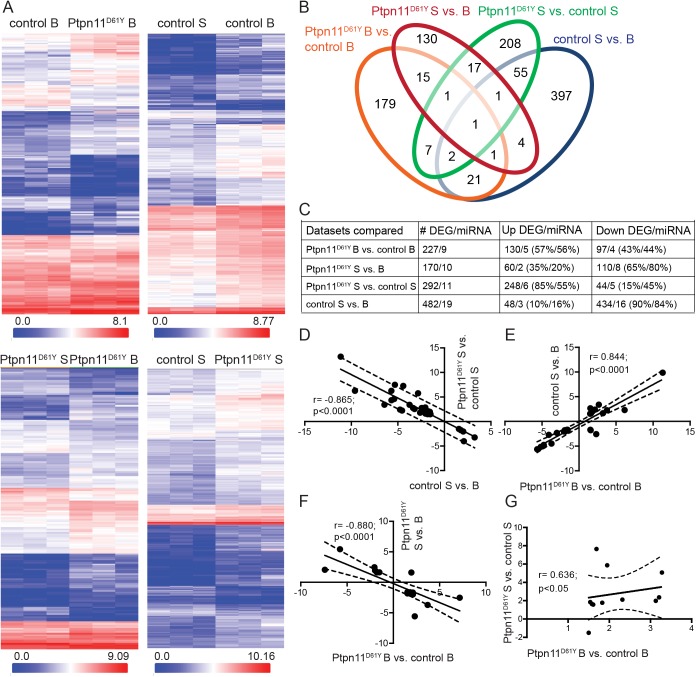
Analysis of DEGs in hippocampi of control and Ptpn11^D61Y^ mice in the basal state and after the stimulation of neuronal activity. **(A)** The heat maps show the expression levels of DEG transcripts for each analyzed dataset. Three biological replicates (columns) per condition are shown for all DEGs (rows). The color code in a logarithmic scale is given for the signal intensities of the DEGs and indicates a low (in blue) or high (red) expression. **(B)** The Venn diagram shows the number of DEGs in each dataset. The intersections indicate the number of genes regulated in two or more datasets. **(C)** The table shows the number of differentially expressed mRNAs and miRNAs as well as the direction of their regulation in each dataset. **(D-F)** Plots show the inter-dependency of the expressional regulations of the DEGs that are commonly regulated between the datasets. Each data point represents one DEG; the x- and y-axis indicate the level of regulation as fold change in a logarithmic scale in the dataset. The correlation coefficient (r) and p-value are indicated in the graphs. The black lines show the best fits; the dashed lines indicate the 95% confidence intervals.

To further investigate the effect of the overactive *Ptpn11*^*D61Y*^ allele on the regulation of neuronal gene expression, we performed an unbiased transcriptome analysis using acute hippocampal slices in the basal state and upon a 10 min pulse application of 4AP and Bic. To allow for changes in the expression of activity-regulated genes, slices were incubated for additional 3h before further processing. At this time point a robust increase of the mRNAs of BDNF and the activity-regulated cytoskeleton-associated protein (Arc) was seen in initial time-course experiments (S3 Fig). We analyzed the expression profiles from 4 different conditions: control slices and slices from Ptpn11^D61Y^ animals, both harvested either upon treatment with vehicle, i.e. in basal conditions (B) or upon stimulation with 4AP and Bic (S). The differential expression analysis was done for four comparisons: 1) Ptpn11^D61Y^ B vs. control B, 2) control S vs. control B, 3) Ptpn11^D61Y^ S vs. Ptpn11^D61Y^ B and 4) Ptpn11^D61Y^ S vs. control S (Fig 8A). For all analyses the filter criteria of ≥1.5/≤-1.5 fold change and p<0.05 for statistical significance were used to select differentially expressed genes (DEGs). All datasets are deposited in the GEO repository (GSE80061). DEGs that passed the filtering for each dataset are summarized in the heat-maps in Fig 9A.

We identified 227 DEGs for the comparison of the transcriptomes of Ptpn11^D61Y^ B vs. control B, thereof 57% of the transcripts were upregulated and 43% were downregulated. As expected, stimulation of neuronal activity led to a significant reprograming of gene expression patterns (control S vs. B). In total 482 DEGs fulfilled the selection criteria, thereof only 10% were upregulated, 90% showed a downregulation. The same treatment in Ptpn11^D61Y^ slices resulted in the regulation of only 170 genes, thereof 35% genes were upregulated and 65% were downregulated. This indicates a much lower complexity of activity-induced expressional regulation in Ptpn11^D61Y^ compared to controls. The transcriptome comparison of Ptpn11^D61Y^ S *vs*. control S revealed 292 DEGs, thereof 85% with higher and 15% with a lower expression in Ptpn11^D61Y^ compared to control, implying that the pronounced activity-induced transcriptional downregulation seen in controls is affected in the mutant (Fig 9B). DEGs coding for microRNAs (miRNAs) were identified in all datasets (Fig 8B). S2 Table contains the full lists of DEGs for all datasets. The regulation of selected regulated transcripts was confirmed by quantitative PCRs (qPCRs) with specific primers. The regulation of all 6 selected coding DEGs (Efna4, Pdgfra, Erbb3, Gabrr1 and Klb) and 5 out of 7 tested mature forms of miRNAs were confirmed (S3 Table).

Next, we analyzed the DEGs commonly regulated in more than 1 datasets (S4 Table). The Venn diagram graphically summarizes their distribution across the datasets (Fig 9C). The largest overlap (59 DEGs) was found in the activity-regulated transcripts in controls (control S vs. control B) and those differentially regulated between mutant and control slices upon activity induction (Ptpn11^D61Y^ S vs. control S). Strikingly, there was a significant negative correlation (Fig 9D) in the regulation of individual DEGs in this group, indicating that activity-driven regulation of these activity-induced genes is attenuated in the hippocampus of Ptpn11^D61Y^ mice. We identified 25 DEGs regulated by activity in the control (control S vs. control B) that were also regulated when comparing Ptpn11^D61Y^ to control in basal state (Ptpn11^D61Y^ B vs. control B). The regulation of individual genes between these conditions showed a significant positive correlation (Fig 9E), which is in line with the role of Ras/MAPK pathway (here upregulated due to the overactivation of Ptpn11) in the control of the neuronal-activity driven gene expression. We identified 18 DEGs that were regulated by neuronal activity in the mutant (Ptpn11^D61Y^ S vs. B) and also between Ptpn11^D61Y^ and control in basal conditions (Ptpn11^D61Y^ B vs. control B). Interestingly, there was a significant negative correlation between individual common DEGs when comparing these two datasets (Fig 9F), which might be due to a compensatory downregulation of some activity-regulated genes in neurons expressing overactive Ptpn11^D61Y^.

### Functional analyses of DEGs

To understand the biological significance of the obtained gene expression profiles, we first analyzed the functional annotations implemented by the Ingenuity Pathway Analysis (IPA) for all DEGs. Annotations that passed the selected threshold (Fischer’s exact test; p<0.01) are listed for the four analyzed datasets in S5.1 Table. In line with multiple cellular roles of Ptpn11, the DEGs regulated in the mutant compared to control (Dataset: Ptpn11^D61Y^ B vs. control B) were linked to cellular division, differentiation, development, morphology, and mobility. Especially high scores (p<10^−4^) were obtained for pathways regulating the cell-fate decision of stem cells. Several pathways linked to morphogenesis and motility were also significantly involved. Eleven DEGs were annotated to the function “olfactory response”; all of them coded for olfactory receptors. Stimulation of control slices (Dataset: control S vs. B) led to a robust and highly significant (p<10^−6^) expressional reprogramming of the genes functionally linked to olfaction (36 DEGs), olfactory response (35 DEGs), signal transduction (51 DEGs) and cell-cell communication (52 DEGs). The vast majority of the genes in these groups of annotations (>30) belong to the superfamily of olfactory receptors, regulated also in the Ptpn11^D61Y^ B vs. control B dataset. Olfactory receptors are an extremely large group (>1.000 murine genes) of 7-transmembrane G-protein coupled receptors implicated in the sensing of odors [31]. Of note, the expression of these genes is not restricted to olfactory tissues and most of the receptors are still “orphan” in the sense of their ligand-specificity, suggesting their function beyond odor sensing [32]. Multiple annotations linked to cellular signaling and immune system were also significantly regulated in this dataset. The functional annotation of the DEGs regulated by activity in mutant slices (Dataset: Ptpn11^D61Y^ S vs. B) was different. Only 9 DEGs with the annotation of the olfactory response were regulated. Interestingly, 7 DEGs coding for microRNAs and multiple protein-coding DEGs were assigned to functional annotations in context of cell migration, proliferation, cancer and inflammation. Finally, the DEGs between stimulated mutant and control slices (Dataset: Ptpn11^D61Y^ S vs. control S) were functionally annotated in the signal transduction (29 DEGs) and the olfactory response (18 DEGs), which likely reflects a failure in the regulation of these genes by neuronal activity in mutant slices. Multiple annotations linked to diseases and inflammation were also linked to this dataset.

The IPA network analysis (S4–S7A Figs; S5.2 Table) and a prediction of the upstream regulators (S4–S7B and S7C Figs; S5.3 Table) were performed to test the coverage of functional networks by the DEGs in the analyzed datasets. For the dataset Ptpn11^D61Y^ B vs. control B, the highest coverage was found for a network with the nodes ERK1/2 and PI3K downstream of the cytosolic estrogen receptor and the membrane tyrosine kinases activated by hormones and growth factors (ERBB3, PDGFR, Insulin, Cg, Lh, FSH, Alp, and LDL complexes; S4A Fig, S5.2 Table). The differentially regulated upstream regulators predicted for this dataset comprised 6 kinases, 4 phosphatases, 2 microRNAs and 26 transcription factors, which suggests that Ptpn11^D61Y^ induces broad changes in the homeostasis of protein phosphorylation and dephosphorylation as well as in the reconfiguration of gene expression in neurons (S4B and S4C Fig; S5.3 Table). A high activation score was predicted for tumor suppressor protein 53 (Fig 9A), which is implied in neurogenesis and in neurite outgrowth, maturation and regeneration [33]. The mechanistic networks of the predicted upstream regulators with the highest score covered signaling downstream of PDGFR and cytokine receptors via the JAK/STAT pathway. The STAT3 pathway was, together with pathways involved in synthesis/metabolism of cholesterol, lipids and steroids, also a significant hit in the Ingenuity canonical pathway analysis (S6 Table).

In the dataset control S vs. B, the induction of activity led to the regulation of genes that were best covered by the IPA functional network containing the pathways activated by insulin and VEGF involving Akt and AMPK signaling, converging on the expressional reprogramming by the regulation of transcription (RNA polymerase II, ENO1, PLOR2F) and chromatin structure (Hdac, Histone H3, PARP10) (S5A Fig, S5.2 Table). The predicted upstream regulators involved 6 kinases and 15 transcription regulators (S5B and S5C Fig; S5.3 Table). The network based on the predicted upstream regulators discerned PAX6, KLK3 as regulation nodes with >4 connections. A significant inactivation was predicted for SPIB and FIGLA, which are transcription factors that were not yet characterized in the brain (S5B Fig; S5.3 Table).

The IPA functional network covering the neuronal activity-regulated genes in Ptpn11^D61Y^ hippocampi (Dataset: Ptpn11^D61Y^ S vs. B) comprised the signaling around Ras/MAPK, Akt, p38MAPK, Jnk and NFκB pathways that are activated by growth factors (NGF, FGF, NT3), hormones (Insulin, POSTN, TAC1, TAC4, estrogen), and chemokines (lectin) (S6A Fig; S5.2 Table). The upstream regulator analyses predicted 3 growth factors, 1 kinase, 3 microRNAs and 15 transcription factors (S6B and S6C Fig; S5.3 Table). When compared to the dataset control S vs. B, the functional network based on the predicted upstream transcriptional regulators comprises less molecules and shows a remarkable decrease in complexity, which is highlighted by the absence of nodes with >4 connections (S6B Fig).

The DEGs significant in the comparison of the transcriptomes of stimulated hippocampi from Ptpn11^D61Y^ and control (Dataset: Ptpn11^D61Y^ S vs. control S) were best covered by the IPA network that comprises the signaling pathways related to cytokines (IL6, IL10, IL13), hormones (relaxin, thyroid hormone), and growth factors (VEGF) signaling as well as to MAPK1/ERK signaling (S7A Fig; S5.2 Table). In this dataset, 11 kinases, 2 microRNAs, 1 phosphatase and 33 transcription factors were predicted as upstream regulators for DEGs (S7B and S7C Fig; S5.3 Table), which implies a pronounced difference in the neuronal activity-induced signaling and gene expression reprogramming between Ptpn11^D61Y^ and control neurons. A complex kinase network containing the NFκB pathway downstream of TNF, interferon and Jnk signaling covers the upstream regulators predicted for this dataset (S7B Fig). A negative activation score was calculated for TBK1, an activator of NFκB signaling, implying its lower activity in Ptpn11^D61Y^ S compared to control S condition (S7C Fig; S5.3 Table). A highly interconnected network of upstream transcriptional regulators was predicted with multiple nodes. The nodes KLK3 and PAX6 appear in this network similarly as for dataset control S vs. B (S7B Fig). However, according to the prediction these nodes are activated in the dataset Ptpn11^D61Y^ S vs. control S but inhibited in the dataset control S vs. B. An activation of STAT1, IRF3 and IRF7 transcription factors was also predicted, which is implied in the interferon production and signaling via the JAK/STAT pathway. These pathways were also predicted in the Ingenuity canonical pathway analysis (S6 Table).

Among the DEGs, numerous transcripts for microRNAs were identified. To understand the biological significance of these regulations we performed miRNA-mRNA target pathway analysis using DIANA miRPath v.2.0 online tool, which predicts significantly enriched target pathways and genes (S7 Table). An increase of miRNAs targeting the Wnt and MAPK signaling pathway was predicted in basal conditions in Ptpn11^D61Y^ compared to control (Ptpn11^D61Y^ B vs. control B), which might reflect the compensatory attenuation of the Ras/MAPK signaling that is overactivated in presence of Ptpn11^D61Y^. Neuronal activity led to a striking downregulation of miRNAs and their predicted target pathways in both control and Ptpn11^D61Y^ neurons. However, the miRNA regulation was, similarly as the regulation of the coding DEGs, strikingly less pronounced in Ptpn11^D61Y^ neurons.

**Fig 10:**
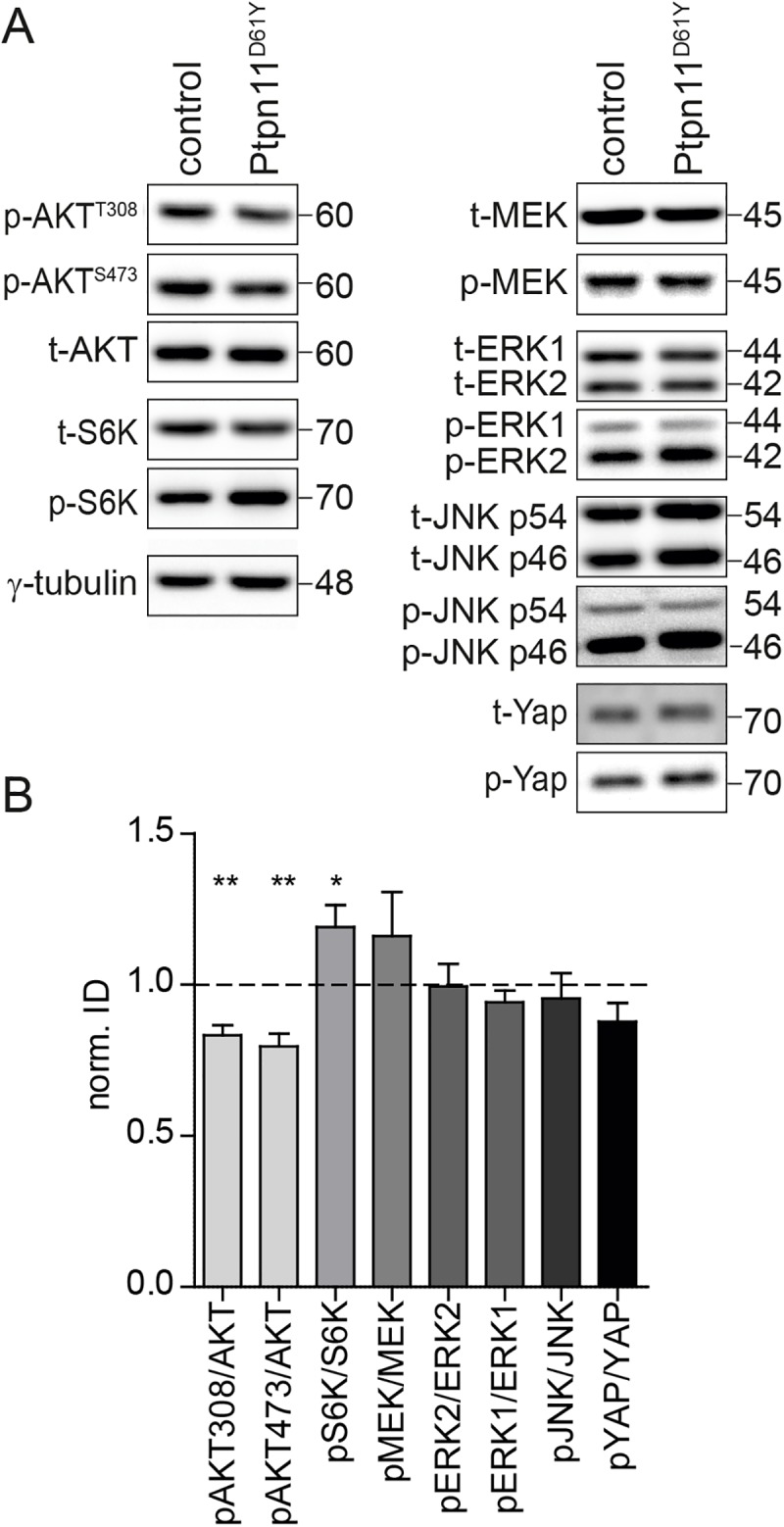
The PI3K-AKT signaling is altered in Ptpn11^D61Y^ brains. (A) Representative Western blots of forebrain homogenate from control and Ptpn11^D61Y^ mice. The phosphorylation level of AKT at the residues Thr308 and Ser473 are reduced, while the phosphorylation of S6K is increased. See S8 Fig for representative images of all proteins analyzed. **(B)** Quantification of the Western blots exemplified in **A**. Data is shown as mean ± SEM and normalized to the expression in controls (n = 3 animals per genotype, unpaired t-test; *p<0.05; **, p<0.01).

To further validate our results from the in silico transcriptome analysis, we performed quantitative Western blots to test the regulation of potentially regulated pathways in the forebrain homogenate of 8 to 12 weeks old Ptpn11^D61Y^ and control animals. We tested the activity of PI3K-AKT signaling by probing immunoblots with antibodies against phospho-AKT isoforms. This analysis confirmed a reduction of AKT phosphorylation at the residues Thr308 and Ser437 (Fig 10; pAKT308/AKT: 83±3% of control; pAKT473/AKT: 80±4% of control, unpaired t-test), which is in line with a dysregulation of PI3K signaling predicted by the in silico analysis. Interestingly, we also observed an increased phosphorylation of S6K on its Thr389 in Ptpn11^D61Y^ (Fig 10; pS6K/S6K: 119±7% of control, unpaired t-test). S6K is a substrate of the activated mTOR complex1, which is regulated by the PI3K/AKT pathway [34]. Thus, these data are in line with a dysregulation of PI3K signaling in Ptpn11^D61Y^. We did not detect any detectable changes in the expression or phosphorylation levels of MEK, ERK1/2, JNK, YAP, p38 and STAT1 (Fig 10, S8 Fig). Importantly, the total expression level of STAT3 was increased in Ptpn11^D61Y^ (S8 Fig; 156±19% of control, unpaired t-test), which was also in agreement with the regulation of JAK/STAT signaling predicted by our in silico analysis.

## Discussion

In this study, we generated a new mouse model expressing the overactivating *Ptpn11*^*D61Y*^ allele to decipher the underlying molecular mechanism of brain dysfunction in RASopathies. The D61Y substitution in *Ptpn11* leads to a strong increase of the basal PTP activity of Ptpn11 [8,10,20]. In humans, *PTPN11*^*D61*Y^ occurs as a somatic mutation in juvenile myelomonocytic leukemia (JMML) with mostly fatal outcome [10]. A global expression of this mutation is lethal in the mouse model [15,20]. Consistently, this mutation has never been observed as a germline mutation in humans, while other germline missense changes at the same codon are compatible with life and lead to a NS phenotype. We have deliberately chosen to use a model based on this particular mutant, because its stronger functional impacts promised that the recognition of significant abnormalities in the nervous system, where patients with NS usually have rather mild impairments, might be easier. In our model, the expression of *Ptpn11*^*D61Y*^ occurs from an endogenous locus, thus corresponding to the expression level of mutant PTPN11 in patients with NS. Since the *Ptpn11*^*D61Y*^ expression in this model is restricted to forebrain excitatory neurons and astroglia it circumvents embryonic and juvenile lethality reported for the constitutive mutants [15,20]. Moreover, this design also allows studying the phenotype linked exclusively to the dysfunction of Ptpn11 in cortical brain circuits and excludes any influences of non-neuronal phenotypes. Recently, cognitive impairment was also reported for mice constitutively expressing the *Ptpn11*^*D61G*^ mutation, which has recurrently been found as a germline mutation in patients with rather severe forms of NS [35]. Although the D61G substitution leads to a smaller increase of the PTP activity than the D61Y described here [8], the impairments in hippocampal learning reported in Ptpn11^D61G^ mice were more severe compared to our observations [11]. This is presumably due to a dysfunction of subcortical circuits in the Ptpn11^D61G^ constitutive model. Systemic effects leading to the perinatal and juvenile death of about 50% of the progeny, could also contribute [12]. The constitutive mouse model expressing the most common NS-associated mutation *Ptpn11*^*N308D*^ showed no alterations in MWM performance and only mild deficits in contextual fear conditioning that is also well comparable with our new model [11]. Altogether the mild cognitive impairment observed in the Ptpn11^D61Y^ mice corresponds well to the clinical neurodevelopmental phenotype in NS patients and justifies the suitability of this model for investigations of the brain-specific pathomechanism of the NS-linked cognitive impairments. Nevertheless, the expression of Ptpn11^D61Y^ in excitatory neurons and astrocytes in our model differs from the global expression in patients with NS. Thus, this mouse model might be more suitable to decipher neuronal molecular and cellular mechanisms than to model the complex pathophysiology in forebrain circuits.

### Normal morphology and synaptogenesis but changes in synaptic glutamate receptor trafficking in Ptpn11^D61Y^ neurons

The expression of *Ptpn11*^*D61Y*^ allele specifically in the forebrain led to a two-fold increased Ptpn11-specific PTP activity as anticipated, but had no effect on mouse viability or gross brain morphology. In this study, we employed cultured hippocampal and cortical neurons allowing the assessment of dynamic processes at the level of individual cells and synapses. The measured increase of Ptpn11-specific PTP activity in neuronal cultures prepared from newborns was comparable to the increase measured in forebrains of adult Ptpn11^D61Y^ mice, justifying our approach. The neuronal morphology and synaptogenesis in culture was not altered, however, we observed differences in the synaptic expression and trafficking of synaptic glutamate receptors. Specifically, the relative surface expression was decreased for GluN2B-containing NMDARs and increased for GluA1-containing AMPA receptors. We suggest that this is due to their abnormal endo- and exocytosis, as we could confirm decreased endocytosis rate of GluA1 in Ptpn11^D61Y^. It is well established that Ras-dependent signaling promotes the externalization of GluA1 at synapses, which is negatively regulated by calcium influx through GluN2B-containing receptors [26]. Thus, the increased GluA1 level we observed is in line with an increased Ras activation in the presence of the overactive Ptpn11^D61Y^ and also with a decreased Ca^2+^ influx through GluN2B. At this point, we can only hypothesize about the mechanism behind the decreased surface expression of GluN2B. Similarly, Ptpn11^D61G^ mutant was reported previously to have a higher neurotransmission [11]. This could explain the observed decrease of GluN2B, as neurotransmission induces an increased endocytosis of synaptic GluN2B-recepors [36]. In line with our data, Lee and colleagues reported a deregulation of surface expression of GluA1 receptors [11]. However, in their case, the receptor expression level was increased and not decreased as we observed. The differences in the experimental setup are likely the reason for this discrepancy. They used an acute (12h) strong overexpression of Ptpn11^D61G^ mediated by viral vectors, while in our case physiological levels of Ptpn11^D61Y^ were expressed during the entire development. Long-term mild overexpression might lead to homeostatic expressional adaptations of these receptors, which are intensively regulated in response to neuronal activity [37–39]. Trafficking of NMDA and AMPA glutamate receptors plays a key role in neuronal plasticity [40]. Therefore a deviation of this process suggested here likely contributes to the aberrant long term-potentiation reported previously for a similar NS model [11].

### Defect in the activity-induced phosphorylation of ERK in Ptpn11^D61Y^ neurons

We observed a stronger increase in the basal phosphorylation of ERK in hippocampal slices of Ptpn11^D61Y^ as compared to the recently published values obtained from slices of Ptpn11^D61G/+^ animals [11]. This is consistent with a higher PTP activity of Ptpn11^D61Y^ mutation described previously and now confirmed here [8] and indicates that the Ptpn11 activity correlates with the level of ERK phosphorylation in the brain. Interestingly, we did not find any significant differences in the relative pERK/ERK levels in full forebrain homogenate of Ptpn11^D61Y^ mice, which is likely due to an upregulation of total ERK in the forebrain of Ptpn11^D61Y^ mice. However, we observed a significant increase of the ratio pERK/ERK in the nuclear fraction prepared from these homogenates that showed no changes in the total ERK abundance. This reveals important differences in the ERK signaling in intact animals and is in line with the observed enhancement of ERK phosphorylation in acute slices and in the nuclei of cultured cells. ERK phosphorylation is strongly regulated by neuronal activity and by growth factors such as BDNF in neurons [41,42]. Having elevated pERK levels already in the basal state, Ptpn11^D61Y^ neurons in high-density cultures and in hippocampal slices failed to increase pERK upon stimulation of neurotransmission or with BDNF. This confirms and expands the previous data about defects in ERK phosphorylation upon theta burst stimulation in hippocampal slices expressing Ptpn11^D61G^ from a viral vector [11]. Moreover, the treatment of Ptpn11^D61Y^ neurons with an inhibitor of MEK1 (a kinase upstream of ERK) fully normalized the basal ERK phosphorylation and rescued the activity-induced increase in pERK, thus confirming that the observed abnormalities in ERK phosphorylation dynamics are indeed mediated by an overactivation of the Ras/MAPK pathway in the Ptpn11^D61Y^ model. Importantly, not only the magnitude but also the temporal course of ERK phosphorylation differed in cells expressing Ptpn11^D61Y^ indicating an aberrant temporal regulation of pERK-dependent gene expression in this NS model.

### Aberrant neuronal activity-dependent gene expression in Ptpn11^D61Y^ neurons

In line with the key role of ERK activation for expressional control of activity-regulated genes, we observed defects in the neuronal activity-induced activation of the BDNF promoter in Ptpn11^D61Y^ neurons. The subsequent gene expression profiling revealed differences in the hippocampal gene expression at basal conditions and after stimulation of neuronal activity in Ptpn11^D61Y^ compared to control mice. A fraction of genes regulated in Ptpn11^D61Y^ under basal conditions overlapped with genes regulated by neuronal activity in controls, which implies an activation of Ptpn11 and its downstream signaling by neuronal activity. This is in line with the well-established role of the Ras/MAPK pathway in neuronal activity-dependent gene expression [18,43]. The network analysis data revealed a regulation of five kinases and four phosphatases, which were linked to signaling related to Ptpn11 in previous studies [44,45] and an increased expression of miRNAs that target Ras/MAPK signaling in Ptpn11^D61Y^ neurons. These regulations might reflect a possible compensatory feedback loop on basal Ras/MAPK signaling in this NS model. Such an adaptive regulation of the Ras/MAPK signaling network could also contribute to the observed defects in the induction of pERK by activity. Increased neuronal activity induced a robust reprogramming of gene expression with an overall tendency towards decreased expression levels in controls. Transcript levels of 90% of DEGs were reduced, while the transcripts of only 10% were elevated. In contrast, the activity-induced regulation of gene expression was largely impaired in Ptpn11^D61Y^: The number of genes regulated by activity is 3-times lower compared to controls. Although the preference for an activity-induced downregulation of genes persists in Ptpn11^D61Y^, the number of downregulated genes is strongly reduced (434 in control vs. 110 in Ptpn11^D61Y^) and their downregulation is less pronounced (see the negative correlation in Fig 9D). The comparison of activity-induced regulation in Ptpn11^D61Y^ and in controls showed that most transcripts are relatively increased in the mutant, thus further supporting a failure of the activity-induced downregulation of gene expression in Ptpn11^D61Y^ neurons.

The functional analysis of DEGs revealed changes in the cellular signaling going beyond a dysregulation of Ras/MAPK axis that has been nearly exclusively discussed in the context of neuronal phenotypes in NS so far [3,11]. Significant changes in the basal JAK/STAT and PI3K/AKT/mTOR signaling in Ptpn11^D61Y^ were predicted in silico and confirmed by quantitative Western blots. These pathways are known targets of Ptpn11 regulation [7,46] and were connected to NS-linked myeloproliferative pathogenesis [47,48]. Our study now suggests these pathways as important targets of Ptpn11 signaling in the brain. The in silico analysis predicted a differential regulation of the JAK/STAT, Jnk and NFκB pathways by neuronal activity in Ptpn11^D61Y^ compared to controls. These pathways contribute to the expressional control of activity-regulated genes [49–51] and Ptpn11 was linked to these pathways by previous studies [3,52,53]. The dysregulation of these pathways and their possible targeting for a therapeutic intervention in the context of NS-associated cognitive impairments should be explored in further studies.

Taken together, this study revealed that the forebrain-restricted expression of the overactive Ptpn11^D61Y^ results in learning impairments and suggests an aberrant neuronal activity-dependent regulation of gene expression as the underlying mechanism. Our data indicate disturbances in several steps of the neuronal activity-induced cellular signaling including the aberrant expression of glutamate receptors on the spine surface, impaired neuronal activity-induced ERK phosphorylation and subsequent regulation of gene expression. Moreover, we provide evidence for changes in JAK/STAT, PI3K, and mTOR signaling. Further, our study suggests that the expression of overactive Ptpn11 in neurons leads to the development of compensatory mechanisms that partially dampen the unconstrained activation of Ras/MAPK signaling in the basal state, whose pathogenic significance has been underestimated, so far. We propose that these mechanisms strongly limit the dynamic range of Ras/MAPK signaling and contribute to the observed blunting of the activity-induced regulation of neuronal gene expression. These findings have important implications for therapeutic considerations regarding neuronal dysfunction in RASopathies. We propose that efficient treatment strategies will probably have to target also JAK/STAT, and PI3K/mTOR pathways and they have to focus on the restoration of the normal dynamics of Ras/MAPK signaling rather than a general inhibition of this cascade.

## Materials and methods

### Animals

Conditional *Ptpn11*^*D61Yfloxed/wt*^ animals (B6.129S6-Ptpn11tm1Toa/Mmjax) [20] were provided by B. G. Neel and T. Araki and bred on C57BL/6J background. For genotyping, the PCR was performed using TGGAGCTGTTACCCACATCA and GCACAGTTCAGCGGGTACTT primers followed by a melting point analysis using the High Resolution Melting and Gene scanning application on the LightCycler 480 (Roche Diagnostics). B6.129S2-Emx1tm1(cre)Krj/J animals [21] were obtained from Jackson Laboratories and genotyped according the provider’s recommendations. Breeding statistics were obtained using to the Python Based Relational Animal Tracking (PyRAT, Scionics Computer Innovation) and analyzed in MS Excel. An average litter size had 6 pups and one breeding couple delivered 5 litters; as calculated from approx. 200 litters of 40 breeding couples.

Breeding of animals, behavioral experiments and experiments using animal material were carried out in accordance with the European Communities Council Directive (2010/63/EU) and approved by the local animal care committees of Sachsen-Anhalt/Germany / Regierungspräsidium Halle Sachsen-Anhalt/Germany (reference number AZ: 42502/2-988 IfN and 42502/2-1295 UniMD).

### Ptpn11-specific tyrosine-phosphatase activity assay

For the Ptpn11-specific protein tyrosine phosphatase activity assay 8 weeks old control and Ptpn11^D61Y^ mice were sacrificed. Forebrain homogenate was centrifuged for 5 min at 1.000xg to obtain the postnuclear S1 supernatant, which was subsequently used for the Ptpn11 immuno-capture and PTP activity measurements. The same experiment was performed using 14 DIV cortical neurons of both genotypes. All components were purchased as a kit DuoSet IC DYC2809 from R&D Systems Inc. and solutions prepared according to manufacturer’s protocols. Colorimetric results were measured at 620 nm wavelength on FLUOstar Omega plate reader by BMG Labtech.

### Antibodies, chemicals and drugs

The following antibodies and chemicals were used for Western Blot (WB), immunocytochemistry (ICC), immunohistochemistry (IHC), recycling assay and Syt1 AB uptake: **mouse antibodies** against Bassoon (mAb7f, IHC 1:1.000; Assay Designs), β-III-tubulin (WB 1:2.000; Sigma-Aldrich), γ-tubulin (1:1000; T5326 Sigma-Aldrich), GluA1 (MAB2263, Recycling Assay, 1:50, Merck Millipore), GluA2 (ICC 1:500; Chemicon/Merck Millipore), panGluA Oyster 550-labeled (ICC: 1:500; Synaptic Systems), Homer1 (ICC 1:500; Synaptic Systems), MAP2 (ICC 1:1.000; Sigma-Aldrich), pERK (M9692, ICC of high density cultures, Sigma-Aldrich) and pERK (M7802, WB 1:500; Sigma-Aldrich), Tau1 (ICC 1:1.000; Millipore), Ptpn11 (SH-PTP2; WB 1:1000; sc-7384 Santa Cruz), **rabbit antibodies** against phosphorylated ERK (#4370, ICC 1:200 in low density cultures; Cell Signaling), ERK (WB: 1:500; Cell Signaling), Bassoon (SAP7f, 1:1.000 [54]), GFP (ICC 1:1.000; Invitrogen), GluN1 (ICC 1:500, Alomone labs), GluN2A (ICC 1:200; Alomone labs), GluN2B (ICC 1:500; Alomone labs), Homer1 (ICC 1:500; Synaptic Systems), Synaptotagmin1 Oyster 550-labeled (Syt1 AB uptake, 1:100; Synaptic Systems), VGAT (ICC and IHC 1:500; Synaptic Systems), VGLUT1 (IHC 1:1.000; Synaptic Systems), MEK1/2 (WB 1:1000; Cell Signaling 9126), ERK1/2 (WB 1:1000; Cell Signaling 9102), AKT (WB 1:1000; Cell Signaling 9272), phospho-MEK1/2 (WB 1:1000; Ser-217/Ser-221, Cell Signaling 9154), phospho-ERK1/2 (WB 1:1000; Thr-202/Thr-204; Cell Signaling 9106), phospho-AKT (WB 1:1000; Ser-473, Cell Signaling 4060; Thr-308, Cell Signaling 2965), p110 (PI3Kinase; WB 1:1000; Cell Signaling 4249), STAT3 (WB 1:1000; Cell Signaling 9139S), phospho-STAT3 (WB 1:1000; Cell Signaling 9145S), FOXO1 (WB 1:1000; Cell Signaling 2880), phospho-YAP (WB 1:1000; Ser 127; Cell Signaling 4911), YAP (WB 1:1000; Cell Signaling 4912), phospho-FOXO1 (WB 1:1000; Ser25; Cell Signaling 9461), JNK (WB 1:1000; Cell Signaling 9252), phospho-JNK (WB 1:1000; Thr 183/Tyr185; Cell Signaling 9251), S6K (WB 1:1000; Cell Signaling 2708) and phospho-S6K (WB 1:1000; Thr389; Cell Signaling 9205), phospho-p38 (WB 1:1000; Thr180/Tyr182; Cell Signaling 9211) and p38 (WB 1:1000; Cell Signaling 8690), **guinea pig antibodies** against Bassoon (ICC 1:500; Synaptic Systems), GAD65 (ICC 1:500; Synaptic Systems), GluA1 (ICC 1:100; Alomone labs), and MAP2 (ICC 1:1.000; Synaptic Systems). **Secondary antibodies** from goat coupled with Alexa 488- (ICC 1:2.000, recycling assay 1:250, IHC 1:500), 647- (ICC 1:2.000) and 680-fluorophore (WB 1:20.000) were purchased from Invitrogen, with Cy3- (ICC 1:2.000; IHC 1:500) and Cy5-fluorophore (ICC 1:2.000) from Jackson ImmunoResearch Laboratories and with CF770 (WB 1:20.000) from Biotium. 4´,6-Diamidin-2-phenylindol (DAPI, 1 μg/ml) was obtained from Sigma-Aldrich. Following **drugs** were used: D-(-)-2-amino-5-phosphonopentanoic acid (APV, 40 μM; Tocris), 6-cyano-7-nitroquinoxaline-2,3-dione disodium (CNQX, 100 μM; Tocris), 4-aminopyridine (4AP, 2.5 mM; Sigma-Aldrich), bicuculline (Bic, 50 μM; Sigma-Aldrich), Brain-derived neurotrophic factor (BDNF, 100 ng/ml; PEPROTECH) and α-[Amino[(4-aminophenyl)thio]methylene]-2-(trifluoromethyl)benzeneacetonitrile (SL327, 1 μM; Tocris).

### Primary culture

Dissociated hippocampal cultures from newborn mice were prepared as described previously [55]. Cells were plated in densities of either 10.000 cells for 5 DIV old neurons, 20.000 cells per coverslip for 14 DIV cells for low density cultures, or 30.000 cells per coverslip for 14 DIV cells for high density cultures.

For the preparation of primary cortical neurons derived from Ptpn11^D61Y^ newborns and their wild-type siblings, postnatal animals (day P0-P1) were killed by decapitation and their cortices were freed of meninges. After treatment with 0.25% trypsin for 20 min at 37°C cells were mechanically triturated in the presence of 100 μl DNase (0.1%), filtered using 70 μm nylon nets (Falcon) and plated in DMEM including 10% of fetal calf serum, 1 mM glutamine and antibiotics (100 U/ml penicillin, 100 μg/ml streptomycin) on poly-D-lysine-coated 6 cm plates with 800.000 cells per plate. After 7–8 hours the medium was changed to Neurobasal A medium supplemented with B27, 1 mM sodium pyruvate, 4 mM Glutamax and antibiotics (100 U/ml penicillin, 100 μg/ml streptomycin). At 4 DIV AraC (Sigma Aldrich) was added to the cells to reach a final concentration of 0.6 μM. All chemicals used for neuronal cultures were obtained from Thermo Fisher Scientific, unless indicated otherwise.

### Immunocytochemistry

Quantitative immunostaining of cultured neurons were done as described earlier [56] with following modifications. Cells were fixed in 4% paraformaldehyde in PBS for 4 min, washed three times in PBS, permeabilized with 0.2% Triton X-100 in PBS for 10 min, washed again and blocked in a PBS buffer containing 2% glycine, 2% BSA, 0.2% gelatin, 50 mM NH_4_Cl. All antibodies were applied in blocking buffer. For quantitative assessment, all coverslips compared in one experiment were processed in parallel using identical antibodies, solutions, and other reagents.

The uptake of Syt1 AB induced by an application of 50 mM KCl for 4 min was done as described before [56]. For surface staining of glutamate receptors neurons were incubated with antibodies recognizing the extracellular domain of GluA1, GluA2, panGluA, GluN1, GluN2A, or GluN2B receptors in Tyrode’s buffer (containing, in mM: 119 NaCl, 2.5KCl, 2 CaCl_2_, 2 MgCl_2_, 30 glucose, 25 HEPES, pH 7.4) for 30 min at 37°C. After Syt1 AB uptake or surface staining of GluRs cells were processed for immunostaining as described above. For GluA1 recycling assay neurons were incubated with GluA1 (GluR1-NT) antibody recognizing the extracellular N-terminus in cell media at 4°C for 30 min to block membrane trafficking. Cells were shortly washed with Tyrode´s Buffer containing 1% BSA and placed back into the original culture plate at 37°C for 30 min to allow for endocytosis. Afterwards, cells were washed again with Tyrode´s buffer and processed for immunostaining as described previously [56].

### BDNFpI+pII-EGFP lentivirus

The lentiviral construct expressing BDNFpI+pII-EGFP was used as described previously [57]. For infection of hippocampal neurons, the viral particles were applied over night at 4 DIV. Infected neurons were used for experiments at 14 DIV.

### Immunohistochemistry

Mice were perfused and brains were cryopreserved and 30–40 μm thick slices were prepared and stained as described previously [58]. Slices were mounted using Fluoromount G DAPI (Southern biotech).

### Image acquisition, analysis and presentation

Overview pictures of single sagittal brain sections were obtained using 2.5X objective. The images were arranged to show a brain overview using Adobe InDesign CS3. Brightness and contrast levels of the presented images were minimally adjusted using either ImageJ software (NIH, http://rsb.info.nih.gov/ij/) or Adobe Photoshop CS3.

Images of stained cultured neurons were acquired on a Zeiss Axio Imager A2 microscope with Cool Snap EZ camera (Visitron Systems) controlled by VisiView (Visitron Systems GmbH) software. In general, single coverslips were acquired using camera settings identically applied to all samples quantified in one experiment. Unspecific background was removed using mathematical subtraction of mean background value measured in three unstained regions of each analyzed image in ImageJ software.

For morphological analysis images were binarized using ImageJ software and Adobe Photoshop CS3, and analyzed using the Sholl analysis plugin in ImageJ software. Longest outgrowing dendrite was measured with the same software.

For analysis of synaptic immunofluorescence synaptic puncta were defined semiautomatically by setting rectangular regions of interest (ROI) with dimensions of about 0.8 by 0.8 μm around local intensity maxima in the channel with the staining for the synaptic marker using OpenView software, written and kindly provided by N.E. Ziv [59]. To decrease the variability of data only synapses on 20 μm long proximal dendritic segments localized 10–30 μm away from the soma were considered. As synaptic marker Bassoon was used for all analyses. For analyses of active synapses and surface and total staining of GluRs receptors only synapses that showed over-threshold staining for both Bassoon and Homer1 were considered. OpenView software was utilized to measure mean IF intensities of synaptic staining.

For the quantification of IF for pERK or BDNFpI+pII-EGFP reporter DAPI staining was used as mask for nuclear ROIs. Masks were generated using Image J software. The integrated density of the nuclear staining was measured using the same software.

Brightness and contrast levels of the presented images were minimally adjusted using either ImageJ software or Adobe Photoshop CS3 and CS6. All panels were arranged in Adobe InDesign and Adobe Illustrator CS3 and CS6.

### Preparation of acute hippocampal slices and neuronal activity induction

Acute hippocampal slices were prepared from 8–11 weeks old control and Ptpn11^D61Y^ mice as described previously [60]. Briefly, mice were anesthetized with isoflurane and euthanized by decapitation according to the institution’s approved protocols. Brains were quickly removed, placed in carbogenated ice-cold artificial cerebrospinal fluid (ACSF, in mM: 125 NaCl, 2.5 KCl, 1.25 NaH_2_PO_4_, 25 NaHCO_3_, 25 D-glucose, 2 CaCl_2_ and 1 MgCl_2_) and hippocampi were dissected. Transverse slices of hippocampus (350 μm thick) were prepared using a McIlwain-type tissue chopper, transferred to small meshed chambers and incubated for recovery in carbogenated (95% O_2_/5% CO_2_) ACSF for 1 h at 37°C.

Thereafter, 3–4 slices originating from the same animal were incubated together in each chamber. Slices were treated with 2.5 mM 4AP and 50 μM Bic or vehicle in ACSF for 10 min and then processed immediately for Western blotting or incubated for additional 3 hours in ACSF prior to RNA isolation. For quantitative western blotting the slices from one chamber were pooled and homogenized using ice-cold lysis buffer containing 50 mM Tris-HCl (pH 7.4), 150 mM NaCl, 0.1% SDS, 1% Triton X-100 and 2 mM EDTA supplemented with Complete Protease inhibitor and PhosSTOP phosphatase inhibitor cocktails (Roche) immediately after the treatment. Lysates were then centrifuged at 14.000 g for 10 min, pellets discarded and colorimetric amido black protein assay was used to determine the protein concentrations of the lysate samples. In each experiment homogenates were prepared from two independently processed chambers per each treatment and genotype. Quantitative immunoblotting was done as described previously [56]. Immunoreactivity was visualized using Odyssey Infrared Scanner (LI-COR Biosciences) and fluorescence intensity of the bands was quantified using Odyssey Software V3.0 (LI-COR Biosciences). As background value, the mean of three empty areas on the membrane was subtracted from each band value and optical density (OD) values were normalized using β-III-tubulin as loading control. The densitometry data were expressed as relative OD. Each sample was loaded in triplicates, resulting in a minimum of 6 values per experiment.

### Preparation of forebrain lysate

To prepare the whole cell lysates, forebrains from 8–12 weeks old male control or Ptpn11^D61Y^ mice were dissected and immediately flash frozen in liquid nitrogen before further processing. Frozen brain samples were homogenized in protein lysis buffer (50 mM Tris-HCl (pH 7.5), 150 mM NaCl, 1% Triton X-100, 0.1% SDS, 2 mM EDTA, 1X protease inhibitor cocktail and 1X phosphatase inhibitor cocktail) and centrifuged to collect the protein-enriched supernatant. Protein concentrations of the lysate samples were determined by BCA assay. Immunoblot analysis was performed as described previously [61]. Equal amounts of cell lysates (20 μg) from control and Ptpn11^D61Y^ (five animals per genotype) were used for the detection of corresponding proteins. Primary antibodies were diluted 1:1000 in 5% nonfat milk (Merck)/TBST (Tris-buffered saline, 0.05% Tween 20). Densitometry analysis of blots was performed using ImageJ software. After background substraction the band intensity was measured. Each blot was repeated two to three times and average was used for analysis. The amount of phosphorylated protein was normalized to the total amount of protein. Unpaired two-tailed t-test was used for statistical analysis in MS Excel.

### Preparation of nuclear fraction from forebrains

Nuclear protein fraction of forebrains from 8–12 weeks old male control or Ptpn11^D61Y^ mice were prepared at 4°C by using CelLytic NuCLEAR extraction kit (Sigma-Aldrich) as per the manufacturer’s recommendations with minor modifications of protocol. Briefly, flash frozen forebrains were quickly washed twice with PBS and homogenized in hypotonic lysis buffer containing DTT, protease and phosphatase inhibitor cocktails. Homogenized samples were centrifuged for 20 min at 11,000xg, supernatant containing cytoplasmic fraction was separated from the crude nuclei pellet. The crude nuclei pellet was re-suspended in extraction buffer (containing DTT, protease and phosphatase inhibitor cocktails), kept on shaker for 30 min followed by centrifugation for 5 min at 21000xg to collect the nuclear protein fraction. Protein concentrations of the lysate samples were determined by BCA assay and equal amount of proteins for each sample were loaded into SDS-PAGE gels for electrophoresis and proteins were transferred to PVDF membranes as described above. The blots were incubated with blocking buffer for 30min followed by primary antibodies (pERK1/2, ERK1/2, β-III tubulin) overnight at 4°C. On the following day, blots were washed thrice with PBS-T (PBS+0.1% Tween-20), incubated with secondary antibodies for 1hr and further washed thrice with PBS-T and twice with PBS. The bands were detected using Odyssey scanner and densitometric analysis was performed by using Odyssey software. Immunoreactivity was normalized to loading control (β-III tubulin) and then the fraction of pERK/ERK was quantified and the data was normalized to control group.

### RNA isolation and quality control

Acute hippocampal slices from animals of both genotypes were prepared and treated in the same way as mentioned above. After 10 min of treatment and incubation in ACSF for additional 3 hours slices were placed in RNAlater RNA stabilization reagent (Qiagen). Total RNA was isolated using the RNeasy Mini Kit (QIAGEN) as per manufacturer’s protocol. RNA integrity (RIN) was determined using ScreenTape R6K kit on the TapeStation2200 (Agilent Technologies); only samples with RIN >7 were further used for microarray hybridization, qPCR and miRNA quantification. The concentration was measured using NanoDrop spectrophotometer at 230, 260 and 280nm (Thermo Scientific, Germany).

### Microarray hybridization, expression data acquisition and analysis

cDNA was prepared from total RNA using a random priming method followed by fragmentation of double-stranded cDNA, labelling and hybridization onto the Affymetrix GeneChip Mouse gene 2.0 ST Arrays with full genome-wide coverage of coding and non-coding transcripts according to the manufacturer’s protocol. Microarrays were scanned with the Affymetrix GeneChip Scanner 3000. Raw data files were corrected for background and imported into Expression Console 1.4.1.46 software (Affymetrix), where the signals were normalized using the quantile method using PLIER algorithm, and mean signals were transformed to log2 scale. The data passed the overall quality assessment done by principal component analysis and other quality metrics. Normalized raw data were analyzed using the software Transcriptome Analysis Console v3.0 (Affymetrix). A fold change of ≥1.5/≤-1.5 and p-value <0.05 (One-way ANOVA) was used as a criterion for the selection of DEGs. Raw array data (.cel files), and processed data (.chp files) are deposited to Gene Ontology Omnibus Database (http://www.ncbi.nlm.nih.gov/geo/; GEO Accession Number GSE80061).

Data mining software Ingenuity Pathway Analysis (IPA; http://www.ingenuity.com/) was used for identification of significantly enriched functional annotations, canonical pathways, upstream regulators and molecular networks for DEGs. IPA implements Fisher's exact test to determine overlap of DEGs with a functional annotation or a canonical pathway. IPA upstream regulator´s analysis function uses the filter criteria of p<0.05 (Fisher’s exact test). To filter the highly significant networks a log(p) transformed score (p-value obtained from Fischer's exact test) higher than 10 was used as threshold.

The miRNA-mRNA target analysis was performed using DIANA miRPath v.2.0 web based software tool (http://www.microrna.gr/miRPathv2), which utilizes previously described DIANA-microT-CDS algorithm [62]. A microT threshold of 0.8 and FDR corrected p-value of 0.01 were used as threshold for predicting the miRNA targets.

### Quantitative real-time PCR (qRT-PCR)

cDNA was synthesized from total RNA samples using RT^2^ First Strand Kit (QIAGEN) according to manufacturer’s instructions and used as template in qRT-PCRs using the RT^2^ SYBR Green qPCR master mix (QIAGEN) and primers specific to the target genes (sequences are listed in S8 Table) performed on the LightCycler480. The expression level of the target genes was normalized to glyceraldehyde-3-phosphate dehydrogenase (GAPDH) as housekeeping transcript. The relative expression level was determined using the relative quantification ΔΔCt method and expressed as fold change to control.

### miRNA quantification

cDNA was synthesized using qScript miRNA cDNA synthesis kit (Quanta Biosciences) according to manufacturer’s protocol and used as template for miRNA amplification using PerfeCTa miRNA assays (Quanta Biosciences) containing specific forward primers and RT^2^ SYBR Green qPCR master mix using the LightCycler480. The expression level of target miRNAs was normalized to the expression of Snord47.

### Behavioral experiments

For all experiments except for Morris Water Maze animals were kept in reverse 12h light/12 h dark cycle. 8–12 weeks old male Ptpn11^D61Y^ and control mice were habituated for at least one week in individual cages. All experiments were performed between 9:00 a.m. and 5:00 p.m.

#### Home cage activity monitoring

Home cage activity was measured as previously described [63]. Mice were monitored and activity was measured for four consecutive days in their home cages using infrared-thermo sensors (Home Cage Activity System, Coulbourn Instruments, Allentown PA), mounted on the top of each cage and interfaced with a computer. Activity was determined from raw values of 15 seconds, which were used to calculate activity periods of 5 min bins. Percentages of activity per hour were calculated from average values of 4 days.

#### Open field exploration (OFT)

Animals were tested for 20 minutes under red light (5 Lux low light conditions) in an open field arena measuring 50 x 50 cm with 35 cm high walls. Exploration was monitored using a video-tracking system (ANY-maze Video tracking system, version 4.50, Stoelting Co, Wood Dale, IL, USA). The distance moved by each mouse and mean speed was measured.

#### Fear conditioning

All tests were performed in a training apparatus containing glass arena and grid floor to deliver foot shock from TSE System, Bad Homburg, Germany. The entire arena was enclosed in a sound-proof cubicle containing speaker, ventilation fan and background noise (70 dB), connected to a computer to measure the freezing behavior, using photo beam system. Mice were tested in a classical auditory cued conditioning paradigm described earlier [64]. Conditioning was done as described previously [65] with minor modifications. Briefly, mice were habituated to training apparatus with six neutral acoustic stimuli (three and three separated with 2 minutes pause; CS- 2.5-kHz, 10 seconds with 20 seconds inter stimulus intervals, ISIs). Training was done 24 hours later with three conditional stimuli (CS+ 10-kHz, 10 seconds with 20 seconds ISIs) each terminating with a 1 second unconditional stimulus (US, scrambled foot shock of 0.4 mA). Fear memory towards shock context was tested 24 hours later. Memory towards different auditory tones was tested in a neutral context using a new standard cage. Freezing behavior (lack of movements except for respiration) was monitored. Fear memory levels were expressed as percentage of time spent freezing during the first two minutes of contextual retrieval in the shock context, cue retrieval in the neutral context and difference between shock and neutral contexts. Cue specific fear memory towards different tones in neutral context was expressed as total freezing levels towards CS+, CS- tone presentations and difference between the two.

With a second batch of mice contextual fear conditioning was also tested. Mice were trained in the fear conditioning chamber after 2 minutes of prior habituation, with a single electric foot shock (0.4 mA, 1 second) followed by 30 seconds interval. This was done for three days and 24 hours after the third training session mice were tested in the shock context for contextual memory retrieval. The fear response was represented as the percentage of freezing during first two minutes of the session.

#### Morris Water Maze (MWM)

Animals were kept under normal light conditions. 13–15 weeks old male Ptpn11^D61Y^ and control mice were habituated for at least one week in individual cages. Spatial learning was assessed in the hidden platform MWM exactly according to a published protocol [11] and analyzed using VideoMot2 and WINTRACK as described by us previously [66].

### Statistical analysis for activity measurements, staining, western blots, quantitative PCRs, and behavioral experiments

All values are shown as mean ± standard error of the mean (SEM). Graphs were plotted and statistics were calculated using Prism 5 software (GraphPad Software, Inc.). Size of groups, number of independent experiments, analyses, and statistical analyses applied are indicated for each result. Analyses used were one sample t-test, unpaired t-test and one-way ANOVA followed by Bonferroni or Dunnett´s multiple comparison test. For all behavioral analyses either t-test (after performing Shapiro-Wilk normality test), one-way ANOVA or two-way ANOVA repeated measures was used followed by post hoc analysis (Scheffe's or Fisher PLSD) using Prism 5 software (GraphPad Software, Inc.), except for MWM where Statview (SAS Institute Inc., Cary, NC) was used. A p-value smaller than 0.05 (p<0.05) was considered significant.

## Supporting information

S1 Fig Total synaptic expression and surface abundance of glutamate receptors. Control and Ptpn11^D61Y^ hippocampal neurons (14 DIV) were stained with antibodies recognizing the subunits GluA2, all GluAs, GluN1 and GluN2 of glutamate receptors. Staining was performed to visualize the surface fraction and total expression of the respective subunits. Synapses were co-labeled with antibodies against Homer1 and Bsn; arrows highlight the co-labeling. For the quantification see **Fig 4B and 4C** and **S1 Table**. Scale bar: 5μm.
(TIF)

S2 Fig Effect of network activity modulation on the nuclear pERK level in low-density hippocampal cultures from Ptpn11^D61Y^ and controls. **(A)** Representative images of DIV14 neurons of both genotypes stained for pERK in the basal state, upon stimulation of neuronal network activity by application of 4AP/Bic for 30 min and after activity silencing using the blockers of glutamatergic transmission APV (40 μM) and CNQX (100 μM) for 30 min. Neurons are labeled with antibodies against MAP2 (neuronal marker), nuclei with DAPI. Scale bar: 10 μm. **(B)** Quantification of the nuclear pERK level in the images as exemplified in **A**. The increase of neuronal activity using 4AP/Bic leads to an elevation of the nuclear pERK level in controls, but fails to do so in Ptpn11^D61Y^ neurons. The silencing of network activity by APV/CNQX treatment shows comparable effects in both, control and Ptpn11^D61Y^ neurons. Data are presented as mean ± SEM and numbers in columns indicate the number of cells analyzed. Statistical assessment was done using one-way ANOVA followed by Bonferroni´s multiple comparison test (***p≤0.0001).
(TIF)

S3 Fig Time course of activity-induced expression of BDNF and Arc. (A) The expression of BDNF was quantified by qPCR in hippocampal slices harvested 1, 3 or 6 h after incubation of slices with ACFS containing 4AP/Bic. Significantly increased BDNF mRNA levels were detected 3 h after stimulation. qPCR was run on samples for each time point and treatment in quadruplicates, significance was tested by one-way ANOVA with Bonferroni’s multiple comparison test; ***p≤0.0001. **(B)** The expression of BDNF and Arc was quantified in treated and control slices 3 h after the treatment. A significant induction was observed for both genes. qPCR was run in triplicates on one sample from treated and untreated slices, significance was tested using unpaired t-test, **p≤0.01, *p≤0.05. Data are presented as mean ± SEM.
(TIF)

S4 Fig Functional analysis of genes with different basal expression levels in hippocampi of control and Ptpn11^D61Y^ mice. **(A)** Molecular network covering the DEGs with highest scores. **(B)** Network of predicted upstream transcription regulators. **(C)** Network of upstream posttranscriptional and posttranslational regulators for all datasets. In all networks, nodes and edges represent genes and gene relationships, respectively. Upregulated and downregulated DEGs are in color code, whereas uncolored nodes represent genes of networks unregulated in the dataset. The legend explains the meaning of color codes, node shapes and edge types. The intensity of the color is proportionate to the fold values of regulation of DEGs.
(TIF)

S5 Fig Functional analysis of genes differentially regulated by activity in hippocampi of control mice. **(A)** Molecular network covering the DEGs with highest scores. **(B)** Network of predicted upstream transcription regulators. **(C)** Network of upstream posttranscriptional and posttranslational regulators for all datasets. In all networks, nodes and edges represent genes and gene relationships, respectively. Upregulated and downregulated DEGs are in color code, whereas uncolored nodes represent genes of networks unregulated in the dataset. The legend explains the meaning of color codes, node shapes and edge types. The intensity of the color is proportionate to the fold values of regulation of DEGs.
(TIF)

S6 Fig Functional analysis of genes differentially regulated by activity in hippocampi of Ptpn11^D61Y^ mice. **(A)** Molecular network covering the DEGs with highest scores. **(B)** Network of predicted upstream transcription regulators. **(C)** Network of upstream posttranscriptional and posttranslational regulators for all datasets. In all networks, nodes and edges represent genes and gene relationships, respectively. Upregulated and downregulated DEGs are in color code, whereas uncolored nodes represent genes of networks unregulated in the dataset. The legend explains the meaning of color codes, node shapes and edge types. The intensity of the color is proportionate to the fold values of regulation of DEGs.
(TIF)

S7 Fig Functional analysis of genes differentially regulated upon activity induction in hippocampi of Ptpn11^D61Y^ and control mice. **(A)** Molecular network covering the DEGs with highest scores. **(B)** Network of predicted upstream transcription regulators. **(C)** Network of upstream posttranscriptional and posttranslational regulators for all datasets. In all networks, nodes and edges represent genes and gene relationships, respectively. Upregulated and downregulated DEGs are in color code, whereas uncolored nodes represent genes of networks unregulated in the dataset. The legend explains the meaning of color codes, node shapes and edge types. The intensity of the color is proportionate to the fold values of regulation of DEGs.
(TIF)

S8 Fig Quantitative Western blot analysis of the activity of Ptpn11-linked signaling in brain lysates from control and Ptpn11^D61Y^ mice. Quantitative Western blots were probed with antibodies against Ptpn11 **(A)**, against components of the PI3K-AKT-S6K/FoxO1 pathway **(B)**, against members of the Ras-Raf-MEK-ERK pathway **(C)**, against JNK **(D)**, p38 **(E)**, YAP **(F)** and STAT1/3 **(G, H)**. Decreased levels of AKT phosphorylated at Thr-308 and Ser-473 and higher levels of phosphorylated Thr389 of S6K were measured in Ptpn11^D61Y^ samples. An increase in the total expression of STAT3 was also detected. A tendency for an increase was observed for p-MEK levels (~15%), however it did not reach statistical significance. No immunoreactivity for phospho-p38Thr-180/Tyr-182, total STAT1 and phosphoSTAT1 and 3 were detectable in the brain samples, even though control samples from pluripotent stem cells, Jurkat or Baf3 cell lines proved the activity of the used antibodies. Homogenates were prepared from 5 mice per genotype; the numbers on the right side of the blots indicate the molecular weight of the relevant marker.
(TIF)

S1 Table Surface and total synaptic expression of glutamate receptors. The statistical comparison of the surface and total synaptic expression of glutamate receptors subunits: The intensity of the specific synaptic immunofluorescence **(S1.1)** and the density of the staining i.e. number of puncta per 20 μm long segments of dendrites **(S1.2)** are shown here. Statistical analyses were calculated using unpaired t-test (ns: not significant, *p<0.05, **p<0.01, ***p<0.001). Data are presented as mean ± SEM.
(XLSX)

S2 Table List of DEGs for all analyzed datasets. Full lists of DEGs for Ptpn11^D61Y^ B *vs*. control B (A), control S *vs*. B (B), Ptpn11^D61Y^ S *vs*. B (C) and Ptpn11^D61Y^ S *vs*. control S (D) including fold changes and ANOVA p-values are provided.
(XLSX)

S3 Table Validation of microarray data by quantitative real-time PCR. Comparison of the qPCR validation and the microarray results: The differential expression of selected genes and miRNAs was validated by qPCR. The results of the microarray analyses are compared to the qPCR quantifications for randomly selected genes. Regulations are expressed as mean fold change. P-values p<0.05 (unpaired t-test) were considered as statistically significant (n = number of independent experiments/biological replicates). The expression levels of mRNAs and miRNAs were normalized to the housekeeping genes GAPDH and Snord47, respectively, using the 2^−ΔΔCT^ method. Fold changes and p-values (ANOVA between the subjects) of the selected DEGs from microarrays are taken from Table 1. Results were expressed as mean fold change ± SEM. *p<0.05, unpaired t-test (n = 5–12).
(DOCX)

S4 Table List of DEGs that are commonly regulated between the datasets. List of DEGs that are commonly regulated for each analyzed dataset are provided.
(XLSX)

S5 Table Results of IPA analysis for all datasets. **(A)** Significantly enriched functional annotations for each dataset. **(B)** Significantly regulated molecular networks. A log(p) transformed score for each network is calculated by IPA tool, where p-value is obtained from Fischer's exact test. A threshold of >10 score was used to filter the significant networks. **C)** Significantly regulated upstream regulators. IPA upstream regulator analysis predicted the significant upstream transcription regulators and kinases/phosphatases using a filter criteria of p<0.05.
(XLSX)

S6 Table Canonical pathways enriched with DEGs by Ingenuity Pathway Analysis. Significantly enriched canonical pathways for Ptpn11^D61Y^ B vs. control B, control S vs. B, Ptpn11^D61Y^ S vs. B and Ptpn11^D61Y^ S vs. control S dataset comparisons were shown in the table. For each canonical pathway, the p-value (probability that each function assigned to the pathway is due to the chance alone) and the DEGs associated with the pathway were shown. Fischer’s exact test p<0.05 was used as filter criterion for the enriched pathways.
(DOCX)

S7 Table Results of miRNA-mRNA target pathway analysis for significantly enriched pathways related to the differentially expressed miRNAs of each dataset. DIANA miRPath v.2.0 tool with the default parameters is used for predicting the target pathways with a p-value threshold of p<0.01.
(XLSX)

S8 Table List of primers used for the validation of genes by qRT-PCR. (DOCX)
