# Correction: Aberrant neuronal activity-induced signaling and gene expression in a mouse model of RASopathy

**DOI:** 10.1371/journal.pgen.1006843

**Published:** 2017-06-16

**Authors:** Franziska Altmüller, Santosh Pothula, Anil Annamneedi, Saeideh Nakhaei-Rad, Carolina Montenegro-Venegas, Eneko Pina-Fernández, Claudia Marini, Monica Santos, Denny Schanze, Dirk Montag, Mohammad R. Ahmadian, Oliver Stork, Martin Zenker, Anna Fejtova

There are errors in the Funding section. The correct funding information is as follows: Funding in the lab of AF comes from Deutsche Forschungsgemeinschaft FE1335 and SFB779, Bundesministerium für Bildung und Forschung GeNeRARe (FKZ 1GM1519B), Leibniz Gemeinschaft LGS Synaptogenetics, Center for Brain Behavioral Studies NN5 (ZS/2016/04/78120). SNR, MRA, OS and MZ are supported by GeNeRARe (FKZ 1GM1519D, B and A). The funders had no role in study design, data collection and analysis, decision to publish, or preparation of the manuscript.

## References

[1] Altmüller F, Pothula S, Annamneedi A, Nakhaei-Rad S, Montenegro-Venegas C, Pina-Fernández E, et al (2017) Aberrant neuronal activity-induced signaling and gene expression in a mouse model of RASopathy. PLoS Genet 13(3): e1006684 doi:10.1371/journal.pgen.1006684 28346493 10.1371/journal.pgen.1006684 PMC5386306

