# Supplementary figures and images for: Aberrant neuronal activity-induced signaling and gene expression in a mouse model of RASopathy
Source: PLoS Genet. 2017 Mar 27;13(3):e1006684. doi: 10.1371/journal.pgen.1006684 (PMC5386306; doi:10.1371/journal.pgen.1006684)

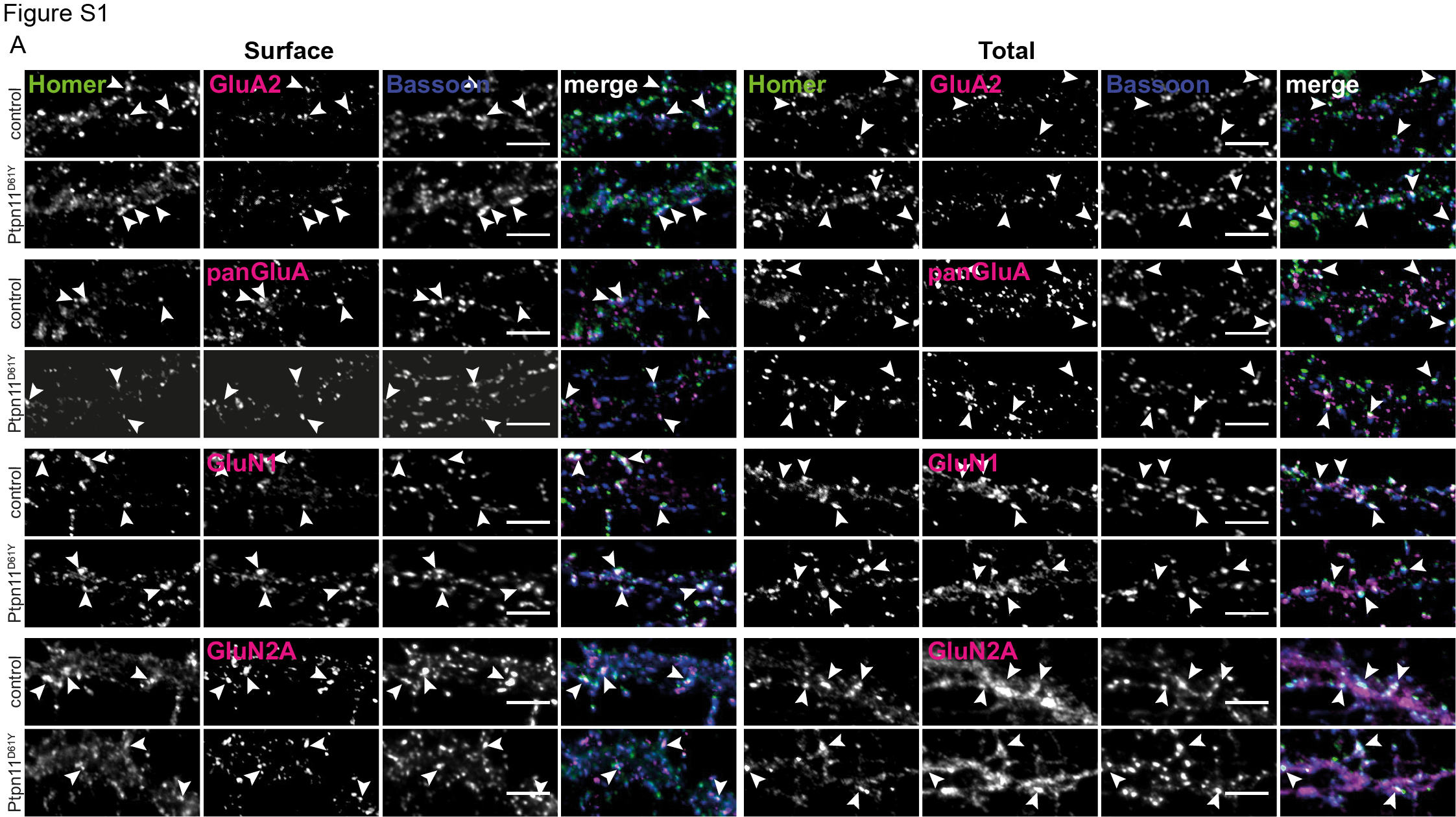

Supplement: S1 Fig — Control and Ptpn11D61Y hippocampal neurons (14 DIV) were stained with antibodies recognizing the subunits GluA2, all GluAs, GluN1 and GluN2 of glutamate receptors. Staining was performed to visualize the surface fraction and total expression of the respective subunits. Synapses were co-labeled with antibodies against Homer1 and Bsn; arrows highlight the co-labeling. For the quantification see Fig 4B and 4C and S1 Table. Scale bar: 5μm. (TIF) [file pgen.1006684.s001.tif]

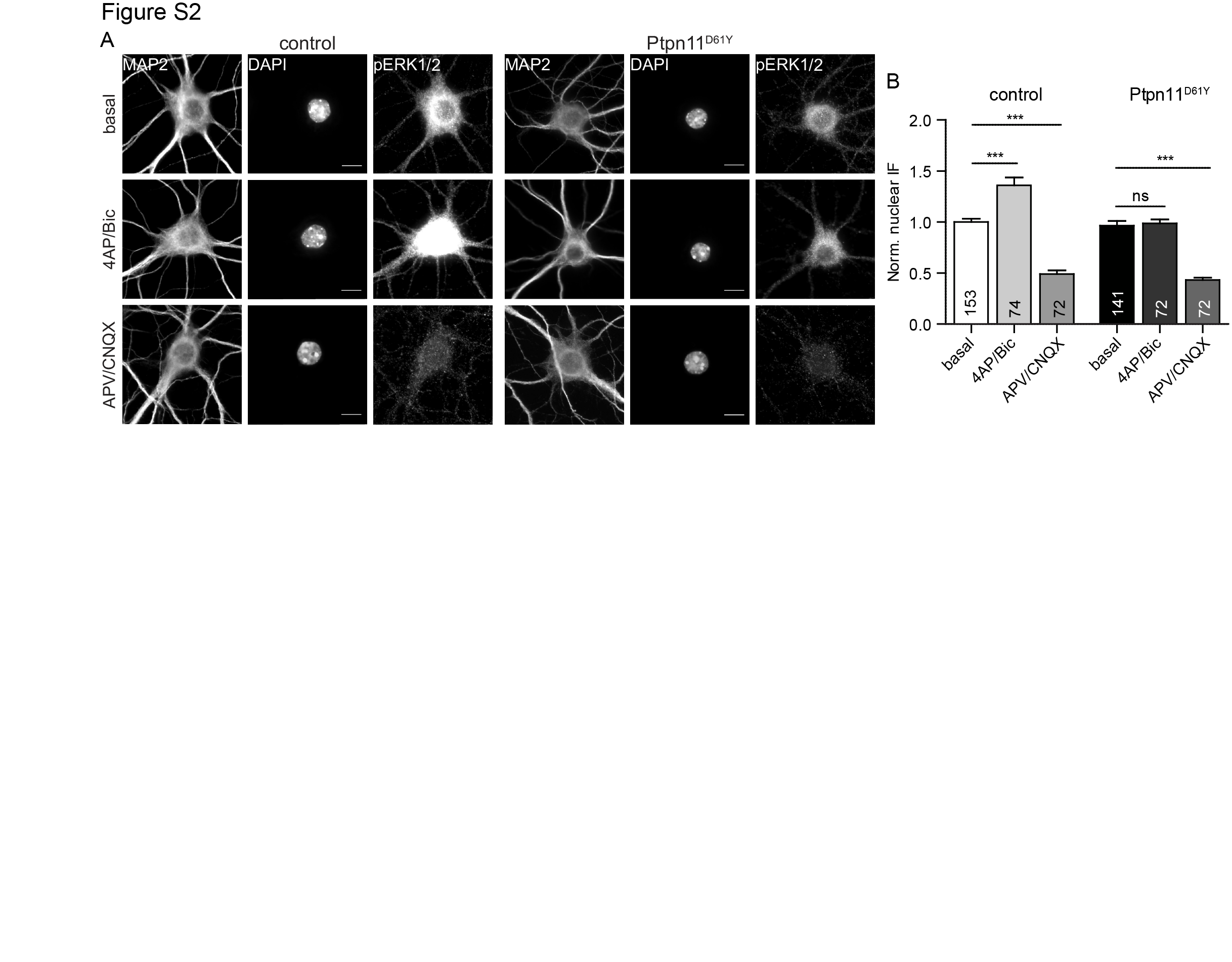

Supplement: S2 Fig — (A) Representative images of DIV14 neurons of both genotypes stained for pERK in the basal state, upon stimulation of neuronal network activity by application of 4AP/Bic for 30 min and after activity silencing using the blockers of glutamatergic transmission APV (40 μM) and CNQX (100 μM) for 30 min. Neurons are labeled with antibodies against MAP2 (neuronal marker), nuclei with DAPI. Scale bar: 10 μm. (B) Quantification of the nuclear pERK level in the images as exemplified in A. The increase of neuronal activity using 4AP/Bic leads to an elevation of the nuclear pERK level in controls, but fails to do so in Ptpn11D61Y neurons. The silencing of network activity by APV/CNQX treatment shows comparable effects in both, control and Ptpn11D61Y neurons. Data are presented as mean ± SEM and numbers in columns indicate the number of cells analyzed. Statistical assessment was done using one-way ANOVA followed by Bonferroni´s multiple comparison test (***p≤0.0001). (TIF) [file pgen.1006684.s002.tif]

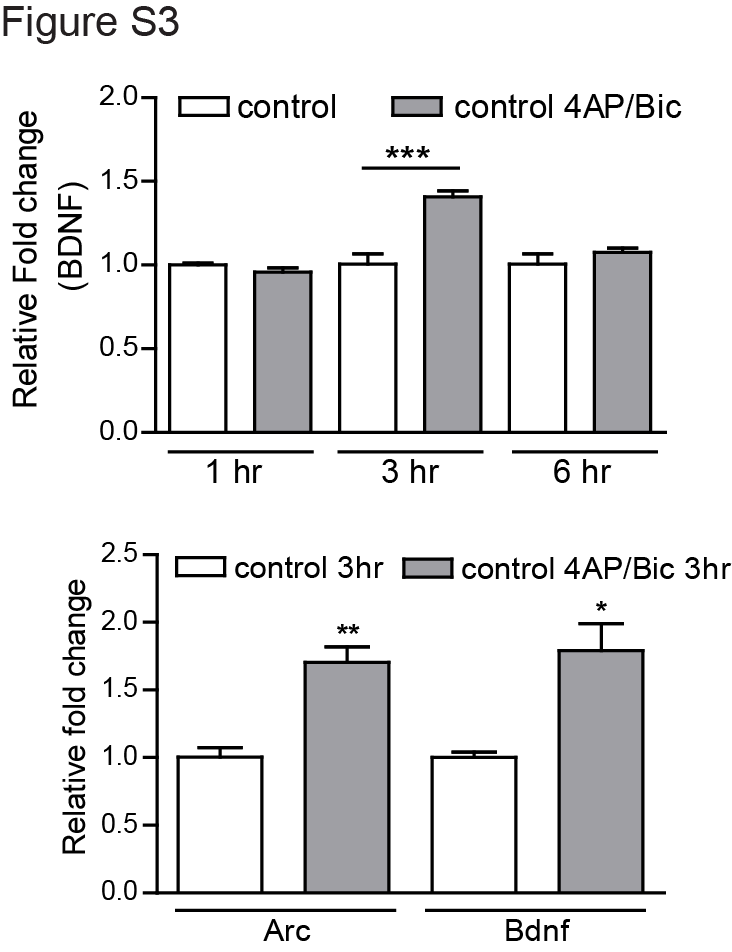

Supplement: S3 Fig — (A) The expression of BDNF was quantified by qPCR in hippocampal slices harvested 1, 3 or 6 h after incubation of slices with ACFS containing 4AP/Bic. Significantly increased BDNF mRNA levels were detected 3 h after stimulation. qPCR was run on samples for each time point and treatment in quadruplicates, significance was tested by one-way ANOVA with Bonferroni’s multiple comparison test; ***p≤0.0001. (B) The expression of BDNF and Arc was quantified in treated and control slices 3 h after the treatment. A significant induction was observed for both genes. qPCR was run in triplicates on one sample from treated and untreated slices, significance was tested using unpaired t-test, **p≤0.01, *p≤0.05. Data are presented as mean ± SEM. (TIF) [file pgen.1006684.s003.tif]

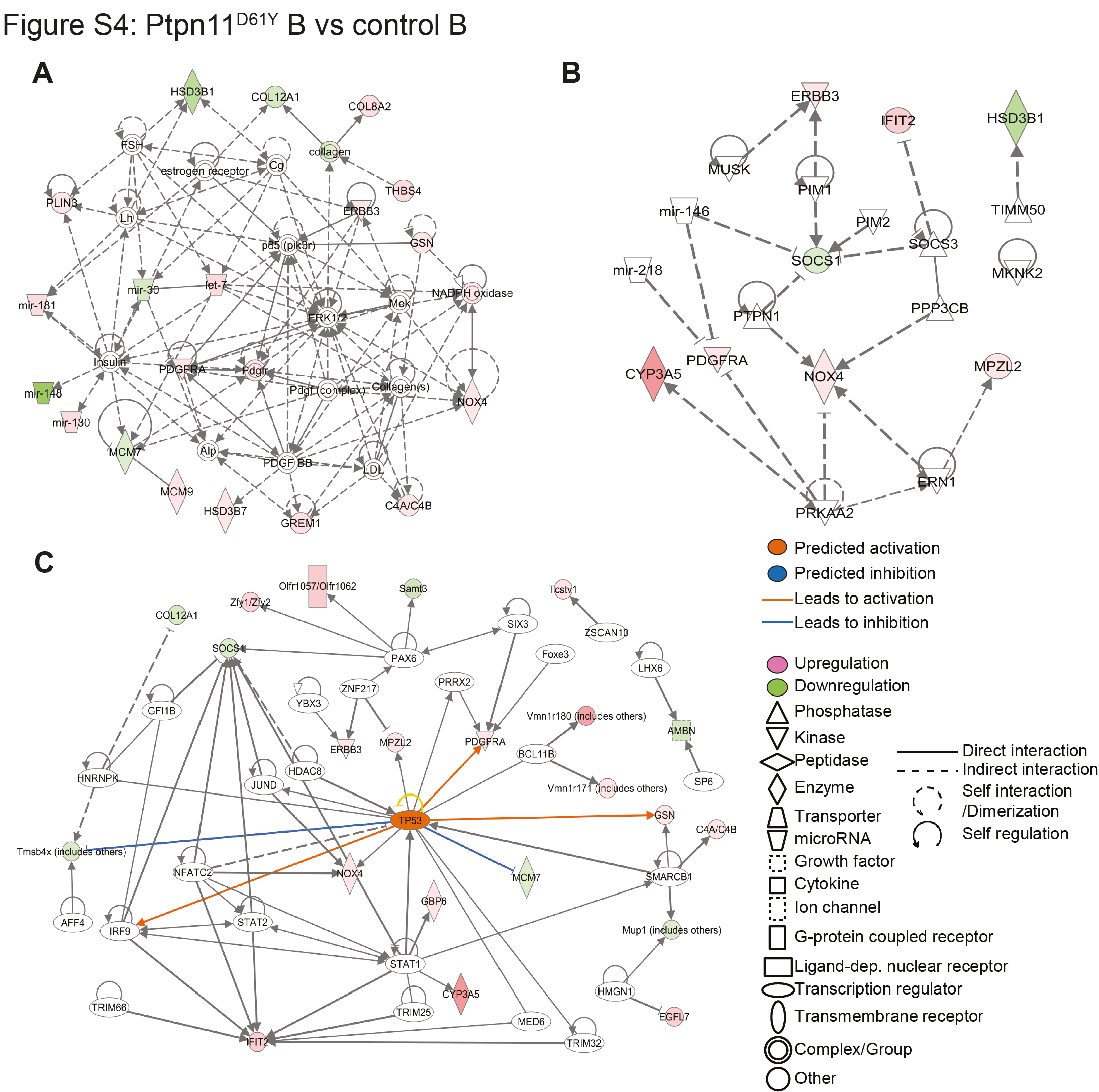

Supplement: S4 Fig — (A) Molecular network covering the DEGs with highest scores. (B) Network of predicted upstream transcription regulators. (C) Network of upstream posttranscriptional and posttranslational regulators for all datasets. In all networks, nodes and edges represent genes and gene relationships, respectively. Upregulated and downregulated DEGs are in color code, whereas uncolored nodes represent genes of networks unregulated in the dataset. The legend explains the meaning of color codes, node shapes and edge types. The intensity of the color is proportionate to the fold values of regulation of DEGs. (TIF) [file pgen.1006684.s004.tif]

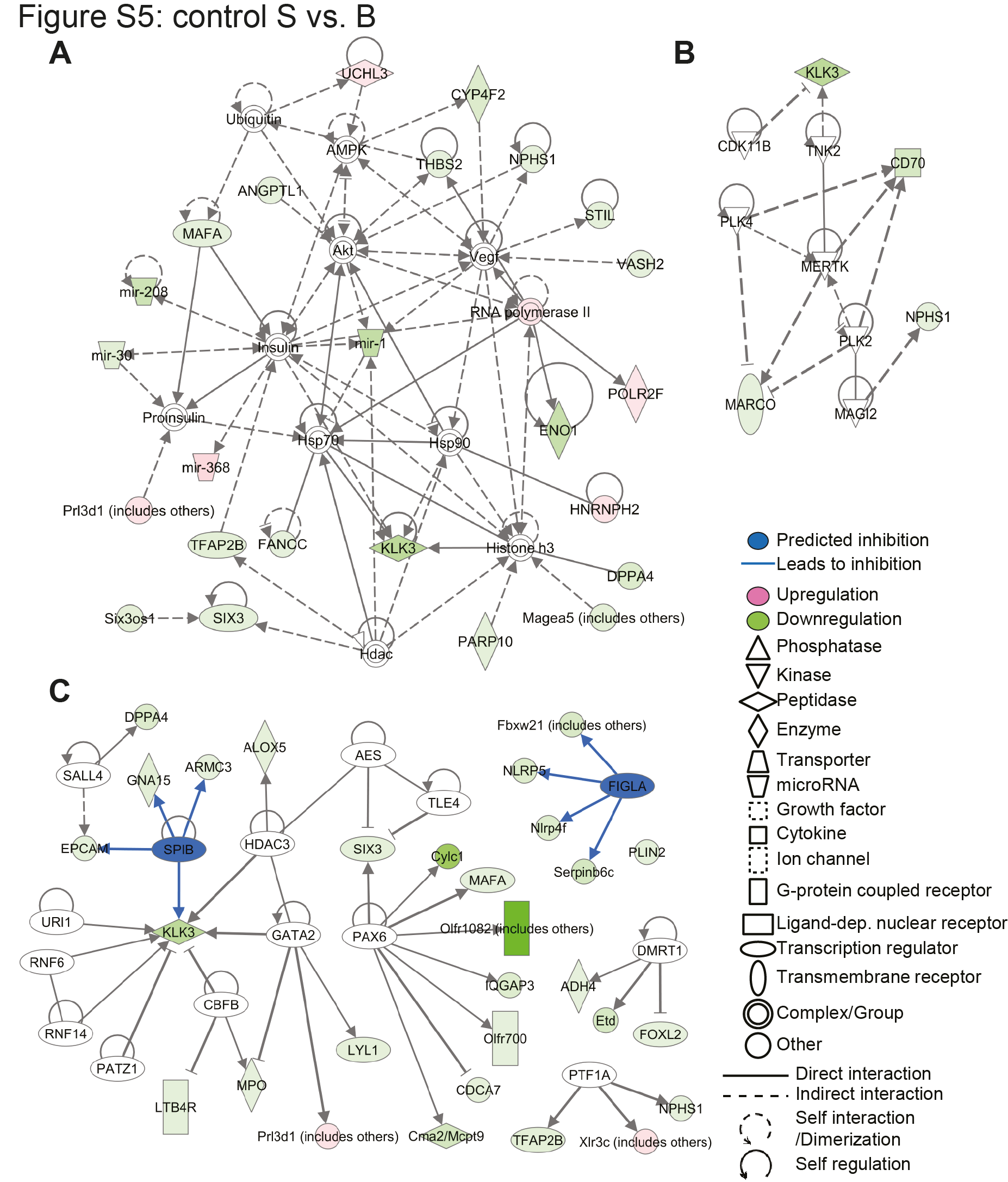

Supplement: S5 Fig — (A) Molecular network covering the DEGs with highest scores. (B) Network of predicted upstream transcription regulators. (C) Network of upstream posttranscriptional and posttranslational regulators for all datasets. In all networks, nodes and edges represent genes and gene relationships, respectively. Upregulated and downregulated DEGs are in color code, whereas uncolored nodes represent genes of networks unregulated in the dataset. The legend explains the meaning of color codes, node shapes and edge types. The intensity of the color is proportionate to the fold values of regulation of DEGs. (TIF) [file pgen.1006684.s005.tif]

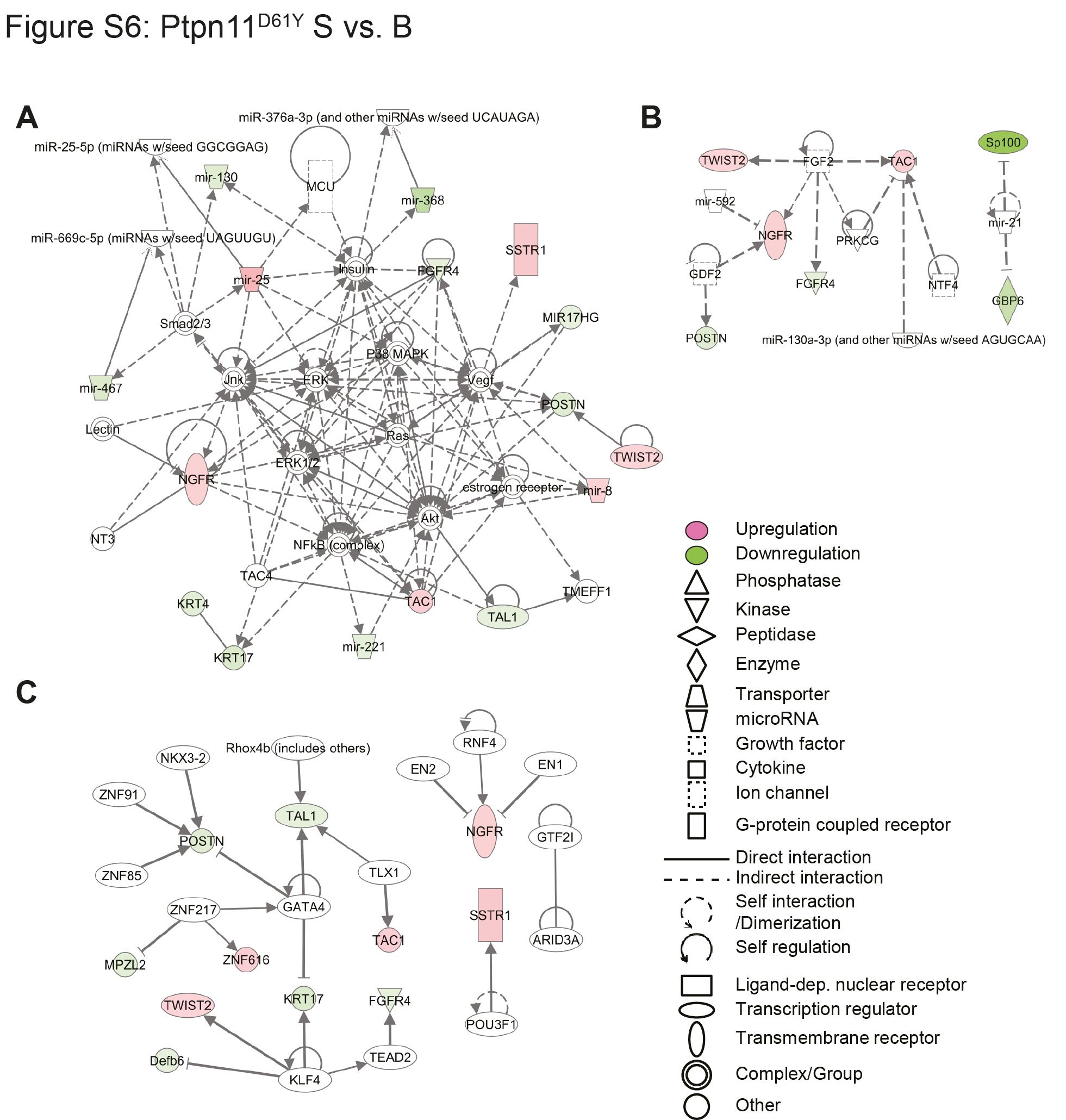

Supplement: S6 Fig — (A) Molecular network covering the DEGs with highest scores. (B) Network of predicted upstream transcription regulators. (C) Network of upstream posttranscriptional and posttranslational regulators for all datasets. In all networks, nodes and edges represent genes and gene relationships, respectively. Upregulated and downregulated DEGs are in color code, whereas uncolored nodes represent genes of networks unregulated in the dataset. The legend explains the meaning of color codes, node shapes and edge types. The intensity of the color is proportionate to the fold values of regulation of DEGs. (TIF) [file pgen.1006684.s006.tif]

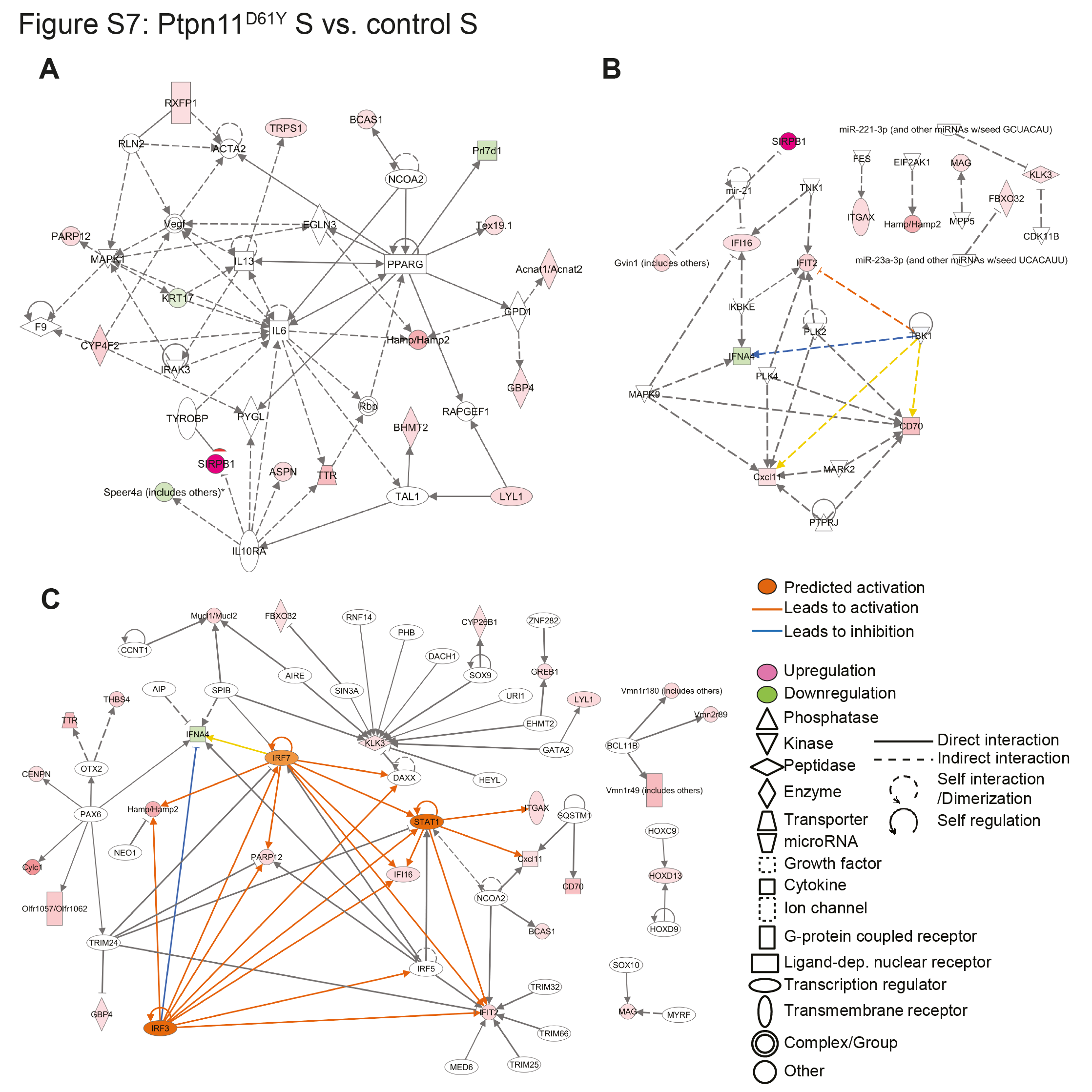

Supplement: S7 Fig — (A) Molecular network covering the DEGs with highest scores. (B) Network of predicted upstream transcription regulators. (C) Network of upstream posttranscriptional and posttranslational regulators for all datasets. In all networks, nodes and edges represent genes and gene relationships, respectively. Upregulated and downregulated DEGs are in color code, whereas uncolored nodes represent genes of networks unregulated in the dataset. The legend explains the meaning of color codes, node shapes and edge types. The intensity of the color is proportionate to the fold values of regulation of DEGs. (TIF) [file pgen.1006684.s007.tif]

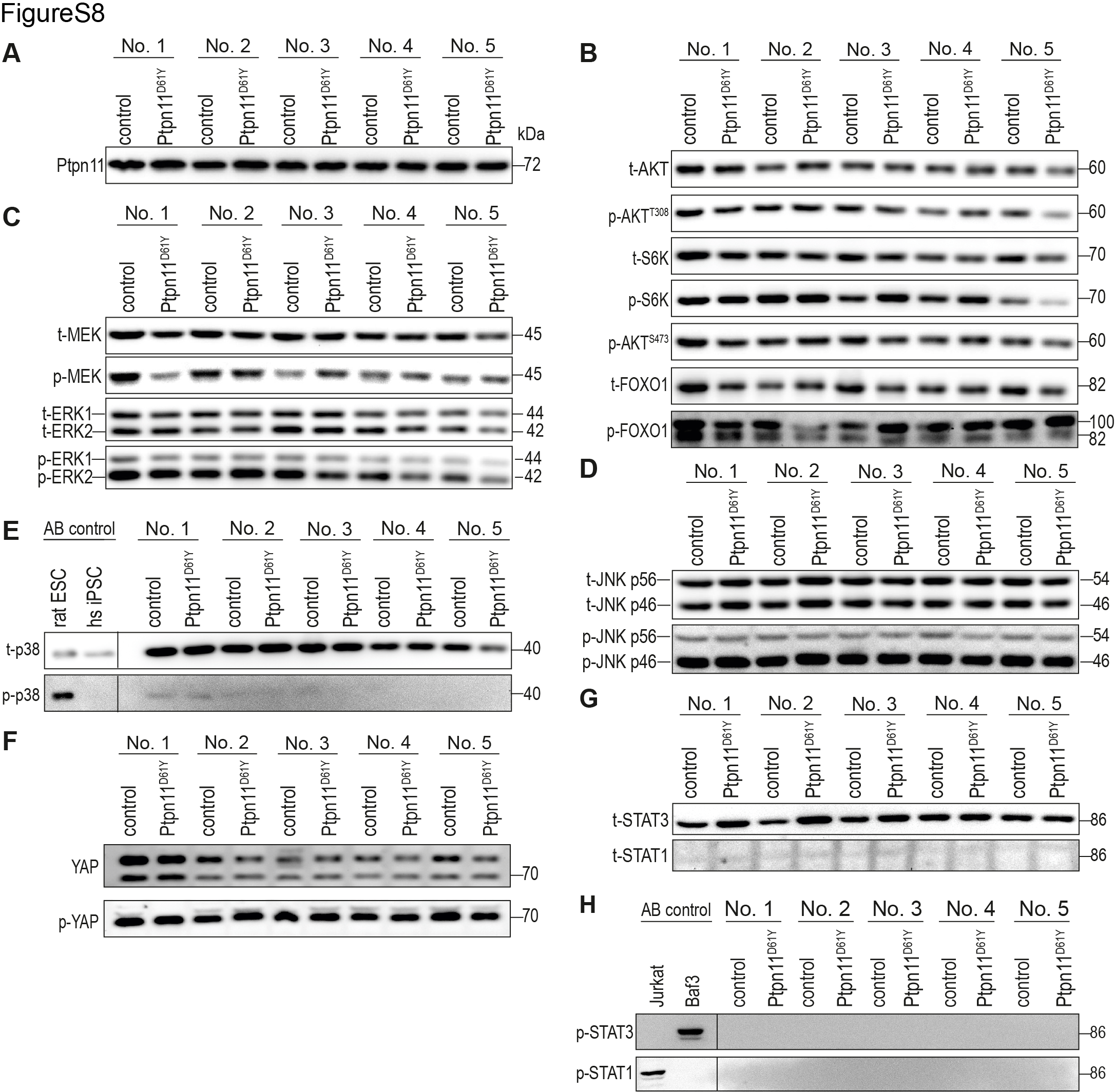

Supplement: S8 Fig — Quantitative Western blots were probed with antibodies against Ptpn11 (A), against components of the PI3K-AKT-S6K/FoxO1 pathway (B), against members of the Ras-Raf-MEK-ERK pathway (C), against JNK (D), p38 (E), YAP (F) and STAT1/3 (G, H). Decreased levels of AKT phosphorylated at Thr-308 and Ser-473 and higher levels of phosphorylated Thr389 of S6K were measured in Ptpn11D61Y samples. An increase in the total expression of STAT3 was also detected. A tendency for an increase was observed for p-MEK levels (~15%), however it did not reach statistical significance. No immunoreactivity for phospho-p38Thr-180/Tyr-182, total STAT1 and phosphoSTAT1 and 3 were detectable in the brain samples, even though control samples from pluripotent stem cells, Jurkat or Baf3 cell lines proved the activity of the used antibodies. Homogenates were prepared from 5 mice per genotype; the numbers on the right side of the blots indicate the molecular weight of the relevant marker. (TIF) [file pgen.1006684.s008.tif]
