# Supplementary material for: Aberrant neuronal activity-induced signaling and gene expression in a mouse model of RASopathy
Source: PLoS Genet. 2017 Mar 27;13(3):e1006684. doi: 10.1371/journal.pgen.1006684 (PMC5386306; doi:10.1371/journal.pgen.1006684)
Supplement: S3 Table — Comparison of the qPCR validation and the microarray results: The differential expression of selected genes and miRNAs was validated by qPCR. The results of the microarray analyses are compared to the qPCR quantifications for randomly selected genes. Regulations are expressed as mean fold change. P-values p<0.05 (unpaired t-test) were considered as statistically significant (n = number of independent experiments/biological replicates). The expression levels of mRNAs and miRNAs were normalized to the housekeeping genes GAPDH and Snord47, respectively, using the 2−ΔΔCT method. Fold changes and p-values (ANOVA between the subjects) of the selected DEGs from microarrays are taken from Table 1. Results were expressed as mean fold change ± SEM. *p<0.05, unpaired t-test (n = 5–12). (DOCX) [file pgen.1006684.s011.docx]

| **Ptpn11^D61Y^ B vs. control B** | | | | | | |
| --- | --- | --- | --- | --- | --- | --- |
|  | **qPCR** | |  | **Microarray** | |  |
| **Gene** | **Fold change** | **p-value (t-test)** | **n** | **Fold change** | **p-value (Anova)** | **n** |
| *Erbb3* | 2.618 | 0.013 | 3 | 1.63 | 0.003 | 3 |
| *Pdgfra* | 1.438 | 0.011 | 3 | 1.62 | 0.009 | 3 |
| *Efna4* | -1.222 | 0.027 | 3 | -1.92 | 0.017 | 3 |
| **control S vs. control B** | | | | | | |
| *Efna4* | -1.802 | 0.002 | 3 | -1.79 | 0.038 | 3 |
| *Gabrr1* | -1.497 | 0.002 | 3 | -2.75 | 0.016 | 3 |
| *Klb* | -1.779 | 0.009 | 3 | -3.21 | 0.022 | 3 |
| **Ptpn11^D61Y^ B vs. control B** | | | | | | |
| *miR-148a* | -1.923 | 0.016 | 3 | -4.07 | 0.048 | 3 |
| *miR-126a* | 1.466 | 0.014 | 3 | 3.12 | 0.020 | 3 |
| *miR-30c-1* | 1.443 | 0.217 | 3 | -1.69 | 0.016 | 3 |
| **control S vs. control B** | | | | | | |
| *miR-206* | 1.301 | 0.084 | 3 | -3.37 | 0.035 | 3 |
| *miR-350* | -1.658 | 0.005 | 3 | -2.06 | 0.011 | 3 |
| *miR-376c* | 1.496 | 0.114 | 3 | 2.09 | 0.028 | 3 |
| **Ptpn11^D61Y^ S vs. Ptpn11^D61Y^ B** | | | | | | |
| *miR-376c* | -1.309 | 0.011 | 3 | -3.45 | 0.021 | 3 |

**S3 Table. Validation of microarray results by qPCR:** Differential expression of selected genes and miRNAs was validated by qPCR. Results are expressed as mean fold change ± SEM. p<0.05 (Student's t-test) was considered as statistically significant (n = number of independent experiments/biological replicates). The expression of mRNAs and miRNAs were normalized to the housekeeping genes Gapdh and Snord47 respectively, using the 2^− ΔΔCT^ method. Fold changes and p-values (ANOVA between the subjects) of the selected genes from microarrays were indicated in the table for the comparison of results from qPCR.
