# Supplementary material for: Aberrant neuronal activity-induced signaling and gene expression in a mouse model of RASopathy
Source: PLoS Genet. 2017 Mar 27;13(3):e1006684. doi: 10.1371/journal.pgen.1006684 (PMC5386306; doi:10.1371/journal.pgen.1006684)
Supplement: S6 Table — Significantly enriched canonical pathways for Ptpn11D61Y B vs. control B, control S vs. B, Ptpn11D61Y S vs. B and Ptpn11D61Y S vs. control S dataset comparisons were shown in the table. For each canonical pathway, the p-value (probability that each function assigned to the pathway is due to the chance alone) and the DEGs associated with the pathway were shown. Fischer’s exact test p<0.05 was used as filter criterion for the enriched pathways. (DOCX) [file pgen.1006684.s014.docx]

| **Ptpn11^D61Y^ B vs. control B** | | | |
| --- | --- | --- | --- |
| **Ingenuity Canonical Pathways** | **p value** | | **Molecules** |
| Mineralocorticoid Biosynthesis | 6.99E-04 | | HSD3B7,HSD3B1 |
| Glucocorticoid Biosynthesis | 8.71E-04 | | HSD3B7,HSD3B1 |
| Androgen Biosynthesis | 1.27E-03 | | HSD3B7,HSD3B1 |
| Complement System | 9.56E-03 | | C4A/C4B,Scgb2b27 (includes others) |
| Nicotine Degradation III | 1.38E-02 | | CYP3A5,UGT2B28 |
| Melatonin Degradation I | 1.38E-02 | | CYP3A5,UGT2B28 |
| Superpathway of Melatonin Degradation | 1.73E-02 | | CYP3A5,UGT2B28 |
| Nicotine Degradation II | 2.03E-02 | | CYP3A5,UGT2B28 |
| STAT3 Pathway | 4.24E-02 | | PDGFRA,SOCS1 |
| Hepatic Fibrosis / Hepatic Stellate Cell Activation | 4.39E-02 | | COL12A1,COL8A2,PDGFRA |
| Bile Acid Biosynthesis, Neutral Pathway | 4.82E-02 | | HSD3B7 |
|  |  | |  |
| **control S vs. B** | | | |
| **Ingenuity Canonical Pathways** | **p value** | | **Molecules** |
| Thio-molybdenum Cofactor Biosynthesis | 0.0101 | | MOCOS |
| Melatonin Degradation III | 0.0101 | | MPO |
| Choline Degradation I | 0.02 | | CHDH |
| Hematopoiesis from Pluripotent Stem Cells | 0.0321 | | IGHM,IGHA1 |
| α-tocopherol Degradation | 0.0397 | | CYP4F2 |
| Primary Immunodeficiency Signaling | 0.0482 | | IGHM,IGHA1 |
|  |  | |  |
| **Ptpn11^D61Y^ S vs. B** | | | |
| **Ingenuity Canonical Pathways** | | **p value** | **Molecules** |
| N-acetylglucosamine Degradation II | | 1.37E-02 | NAGK |
| CMP-N-acetylneuraminate Biosynthesis I (Eukaryotes) | | 1.70E-02 | NAGK |
| Regulation of the Epithelial-Mesenchymal Transition Pathway | | 2.43E-02 | TWIST2,mir-200,FGFR4 |
| STAT3 Pathway | | 2.60E-02 | NGFR,FGFR4 |

| **Ptpn11^D61Y^ S vs. control S** | | |
| --- | --- | --- |
| **Ingenuity Canonical Pathways** | **p value** | **Molecules** |
| Complement System | 1.88E-02 | C4A/C4B,ITGAX |
| Methionine Salvage II (Mammalian) | 1.90E-02 | BHMT2 |
| α-tocopherol Degradation | 2.53E-02 | CYP4F2 |
| Retinoic acid Mediated Apoptosis Signaling | 3.22E-02 | PARP12,IFNA4 |
| Activation of IRF by Cytosolic Pattern Recognition Receptors | 4.06E-02 | IFIT2,IFNA4 |
