# Supplementary material for: Aberrant neuronal activity-induced signaling and gene expression in a mouse model of RASopathy
Source: PLoS Genet. 2017 Mar 27;13(3):e1006684. doi: 10.1371/journal.pgen.1006684 (PMC5386306; doi:10.1371/journal.pgen.1006684)
Supplement: S8 Table — (DOCX) [file pgen.1006684.s016.docx]

| **Gene**  **Symbol** | **Gene name** | **Forward primer** | **Reverse primer** |
| --- | --- | --- | --- |
| ***Erbb3*** | v-erb-b2 erythroblastic leukemia viral oncogene homolog 3 | 5' TTGCCTACAGGAACGCTTACCCG 3′ | 5' ACCCCCCAAAACCGCAGAATC 3′ |
| ***Pdgfra*** | Platelet derived growth factor receptor, alpha polypeptide | 5' AGCGCTGTAGGGGACCCGGAG 3' | 5' GGCCCTGTGAGGAGACAGCTGAG 3' |
| ***Efna4*** | Ephrin A4 | 5' CAGCGCTACACACCCTTCCC 3' | 5' GTGATGACCCGCTCTCCTTG 3' |
| ***Gabrr1*** | Gamma-aminobutyric acid (GABA) C receptor, subunit rho 1 | 5' GAATCTATGTTGGCTGTCCAGA 3' | 5' TGGTGTGGAATTCTTGAATGAG 3' |
| ***Klb*** | Klotho beta | 5' CAGAGAAGGAGGAGGTGAGG 3′ | 5' CAGCACCTGCCTTAAGTTGA 3′ |
| ***Gapdh*** | Glyceraldehyde-3-phosphate dehydrogenase | 5' ACCACAGTCCATGCCATCAC 3' | 5' CACCACCCTGTTGCTGTAGCC 3' |

**S8 Table: List of primers used for the validation of genes by qRT-PCR.**
